# Supplementary material for: Identification of Chemical Markers for the Discrimination of Radix Angelica sinensis Grown in Geoherb and Non-Geoherb Regions Using UHPLC-QTOF-MS/MS Based Metabolomics
Source: Molecules. 2019 Sep 30;24(19):3536. doi: 10.3390/molecules24193536 (PMC6804141; doi:10.3390/molecules24193536)
Supplement: Supplementary file 1 [file molecules-24-03536-s001.pdf]

Characteristic Chemical Markers for the Discrimination of Radix *Angelica sinensis* between Geoherb and non-Geoherb Regions by UHPLC-QTOF-MS/MS Based on Metabolomics Approach

Kaixue Zhang [1], Menglin Yan [2], Shu Han [3], Longfei Cong [2], Liyao Wang [1], Liu Zhang [1], Lili Sun [1], Haiying Bai [1], Guanhua Wei [1], Hong Du [3], Min Jiang [2], Gang Bai [2], Zhigang Yang [1,*]

[1] School of Pharmacy, Lanzhou University, Lanzhou 730000, China; zhangkx2018@lzu.edu.cn (K.Z.); lywang17@lzu.edu.cn (L.W.); zhangliu17@lzu.edu.cn (L.Z.); sunll2018@lzu.edu.cn (L.S.); baihy14@lzu.edu.cn (H.B.); weigh2018@lzu.edu.cn (G.W.)

[2] College of Pharmacy, Nankai University, State Key Laboratory of Medicinal Chemical Biology, Tianjin 300350, China; 15736875593@163.com (M.Y.); 2120181290@mail.nankai.edu.cn (L.C.); minjiang@nankai.edu.cn (M.J.); gangbai@nankai.edu.cn (G.B.)

[3] School of Chinese Materia Medica, Beijing University of Chinese Medicine, Beijing 100029, China; hs361015@163.com (S.H.); duhong@vip.163.com (H.D.)

* Correspondence: yangzg@lzu.edu.cn (Z.Y.); Tel.: +86-931-8915202

Table S1 Origin of the RAS samples

Sample No	origin
1	Xintian Village, Banqiao Town, Enshi City, Hubei Province
2	Xintian Village, Banqiao Town, Enshi City, Hubei Province
3	Shiyao County, Enshi City, Hubei Province
4	Shiyao County, Enshi City, Hubei Province
5	Zanzha Village, Huzhu County, Haidong City, Qinghai Province
6	Yahe Village, Huzhu County, Haidong City, Qinghai Province
7	Heer Village, Huzhu County, Haidong City, Qinghai Province
8	Yaojiagou Village, Huzhu County, Haidong City, Qinghai Province
9	Zelin Village, Huzhu County, Haidong City, Qinghai Province
10	North Hill, Huzhu County, Haidong City, Qinghai Province
11	Pingba Town, Wenshan City, Yunnan Province
12	Pingba Town, Wenshan City, Yunnan Province

13	Lijiang City, Yunnan Province
14	Pingba Town, Wenshan City, Yunnan Province
15	Sanjiacun supermarket, Lijiang City, Yunnan Province
16	Huichuan Town, Weiyuan County, Gansu Province
17	Tianjiagou Town, Weiyuan County, Gansu Province
18	Huichuan Town, Weiyuan County, Gansu Province
19	Zhuangjiahan Cooperative, Lintan County, Gansu Province
20	Zhuangjiahan Cooperative, Lintan County, Gansu Province
21	Zhuangjiahan Cooperative, Lintan County, Gansu Province
22	Lintan County, Gansu Province
23	Gannan City North Community, Lintan County, Gansu Province
24	Changchuan Town, Lintan County, Gansu Province
25	Jinzhong Town, Zhang County, Gansu Province
26	Chiba Village, Chiba Town, Longnan City, Gansu Province
27	Maying Town, Longnan City, Gansu Province
28*	Tianjiabao Village, Xizhai Town, Min County, Gansu Province
29*	Guanshang Village, Xizhai Town, Min County, Gansu Province
30*	Zhongzhai Village, Shili Town, Min County, Gansu Province
31*	Wajie Village, Shendu Town, Min County, Gansu province
32*	Maliantan Village, Weixin Town, Min County, Gansu Province
33*	Hetuo Village, Hetuo Town, Min County, Gansu Province
34*	Shangdiema Village, Qingshui Town, Min County, Gansu Province
35*	Tianjiabao Village, Xizhai Town, Min County, Gansu Province
36*	Zhongzhai Village, Shili Town, Min County, Gansu Province
37*	Maliantan Village, Weixin Town, Min County, Gansu Province
38*	Hetuo Village, Hetuo Town, Min County, Gansu Province
39*	Guanshang Village, Xizhai Town, Min County, Gansu Province
40*	Liujiagou Village, Puma Town, Min County, Gansu Province

*: represents RAS samples from geoherb region.

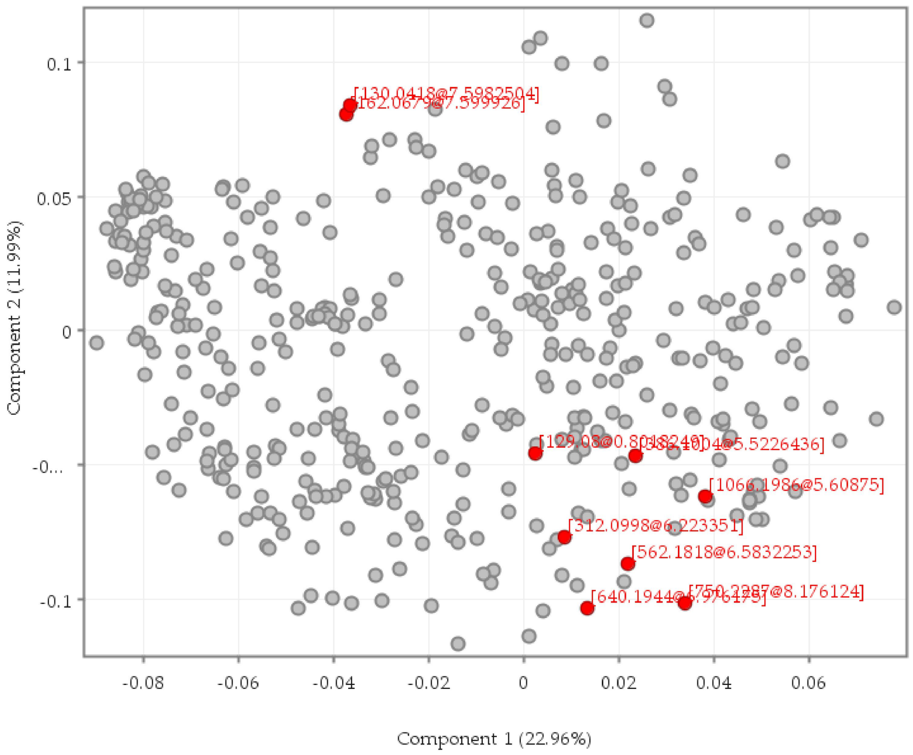

Figure S1 The PCA loadings, highlighting the markers **M1**-**M9**

Figure S2 Extracted ion chromatogram at m/z 387.1074 (positive mode) of RAS from geoherbs (a) and non-geoherbs (b).

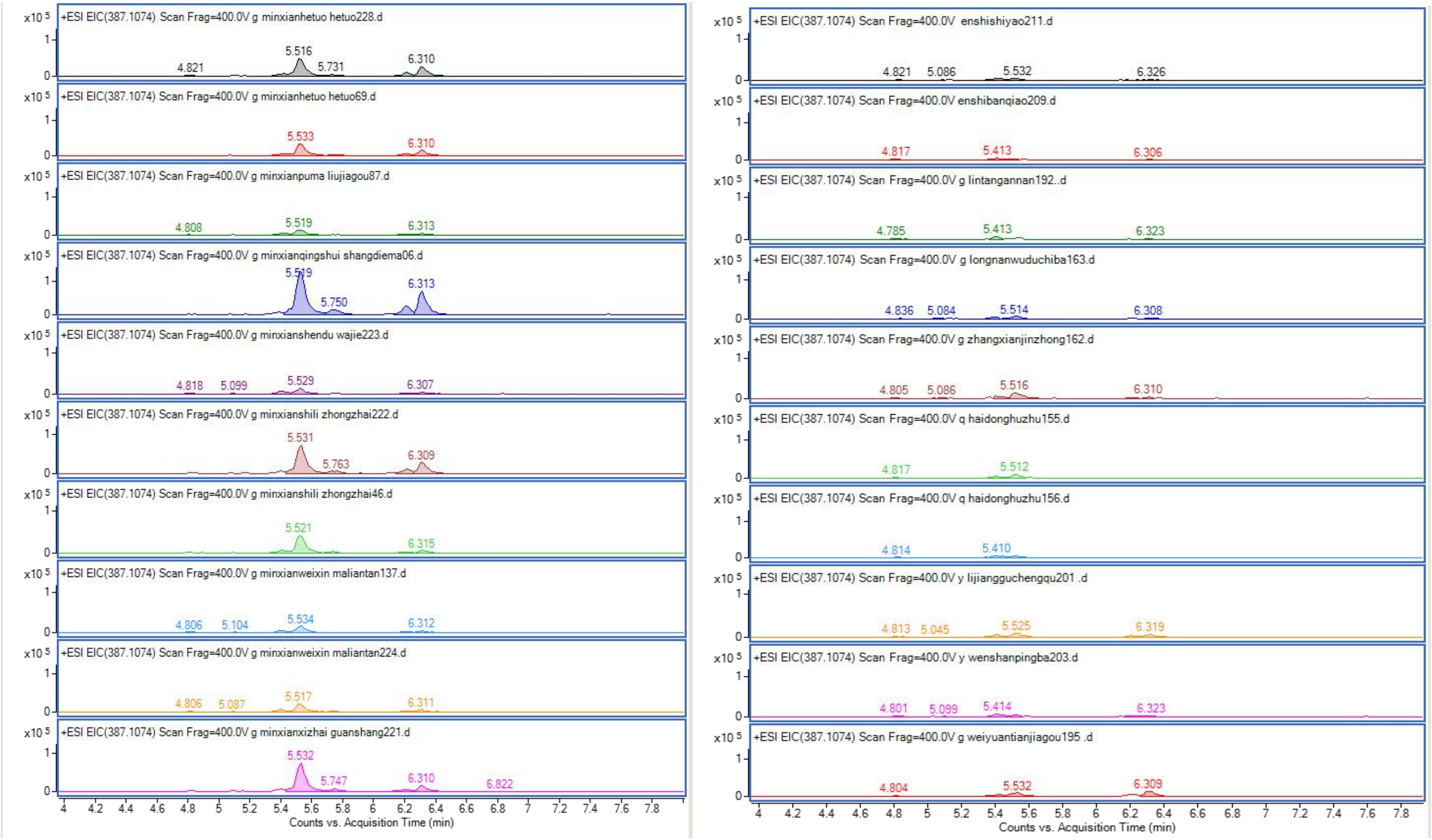

a

b

Figure S3 The MS/MS spectra of compounds detected in RAS.

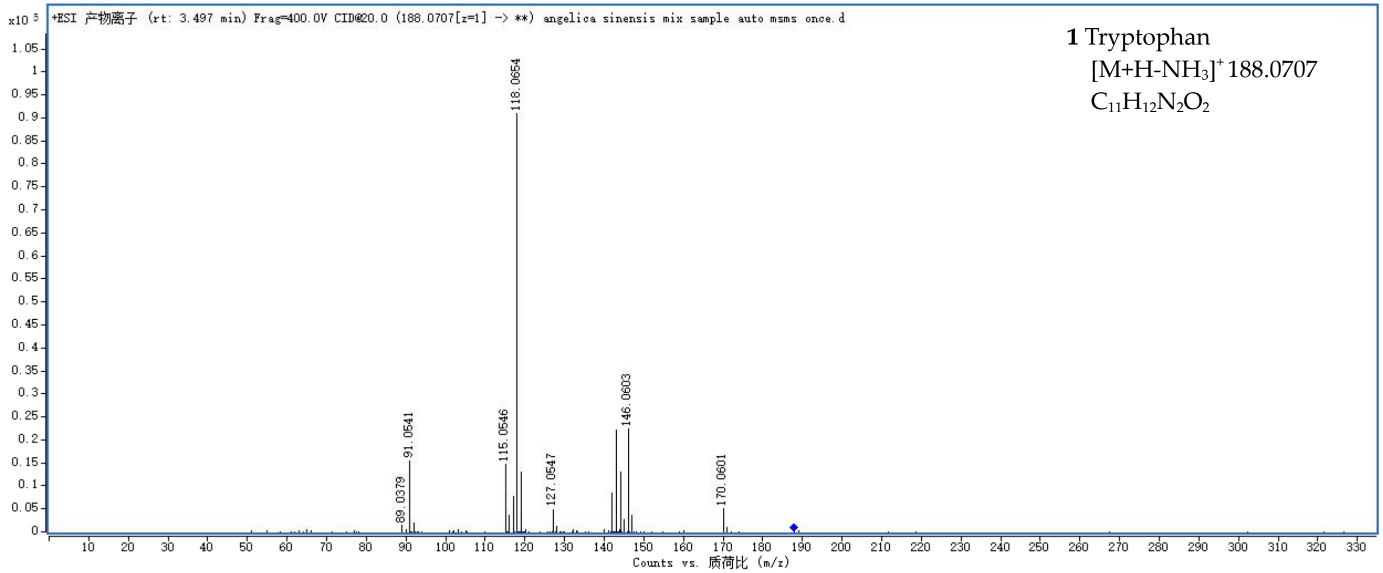
+ESI 产物离子 (rt: 3.497 min) Frag=400.0V CID@20.0 (188.0707[z=1] -> **) angelica sinensis mix sample auto msms once.d
x10 5
1 Tryptophan
[M+H-NH3]+ 188.0707
C11H12N2O2
118.0654
146.0603
91.0541
115.0546
127.0547
170.0601
89.0379
Counts vs. 质荷比 (m/z)

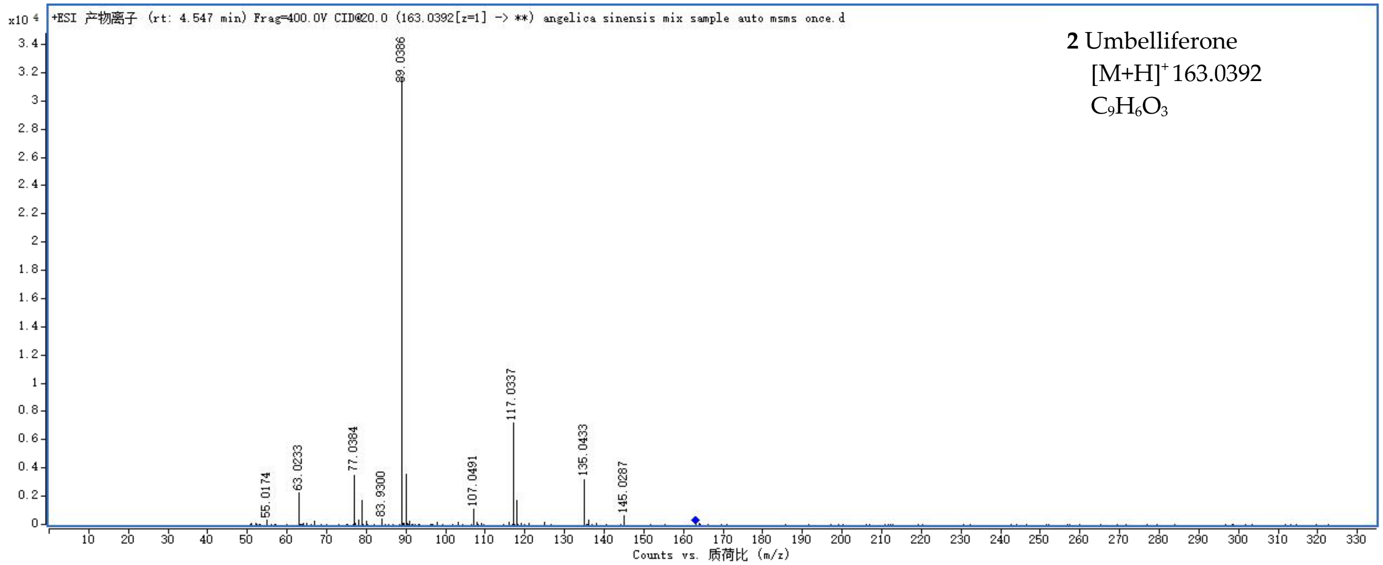
+ESI 产物离子 (rt: 4.547 min) Frag=400.0V CID@20.0 (163.0392[z=1] -> **) angelica sinensis mix sample auto msms once.d
x10 4
2 Umbelliferone
[M+H]+ 163.0392
C9H6O3
89.0386
117.0337
77.0384
135.0433
63.0233
145.0287
107.0491
83.9300
55.0174
Counts vs. 质荷比 (m/z)

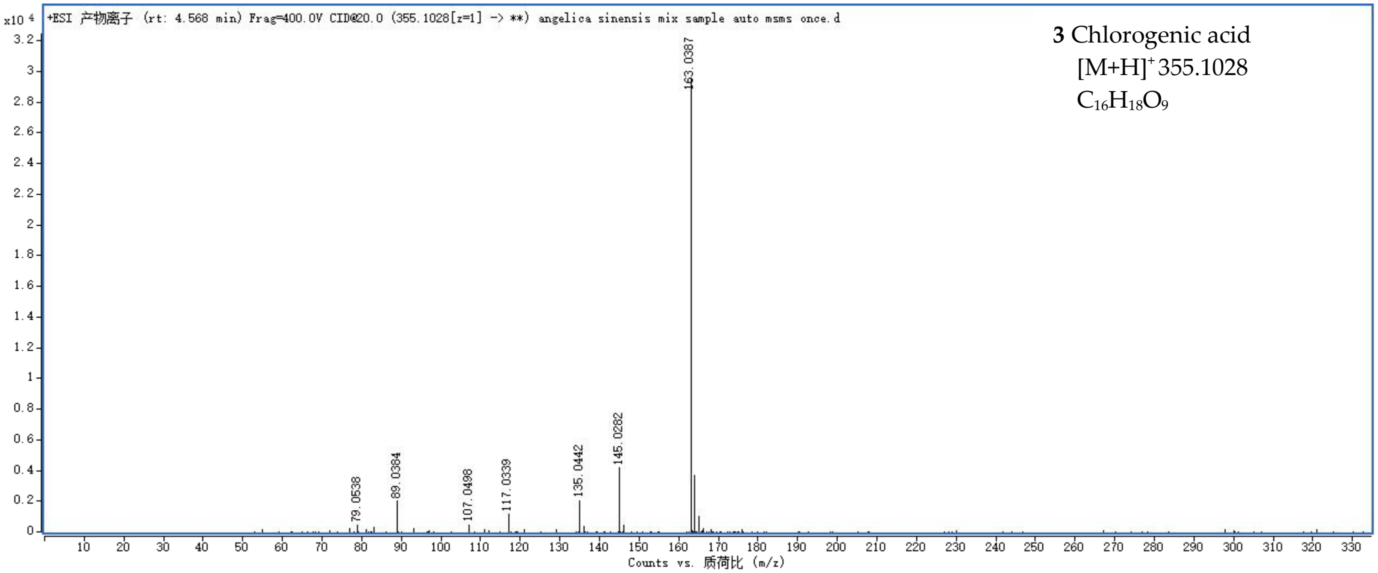
+ESI 产物离子 (rt: 4.568 min) Frag=400.0V CID@20.0 (355.1028[z=1] -> **) angelica sinensis mix sample auto msms once.d
x10 4
3 Chlorogenic acid
[M+H]+ 355.1028
$C_{16}H_{18}O_9$
163.0387
145.0282
135.0442
117.0339
107.0498
89.0384
79.0538
Counts vs. 质荷比 (m/z)

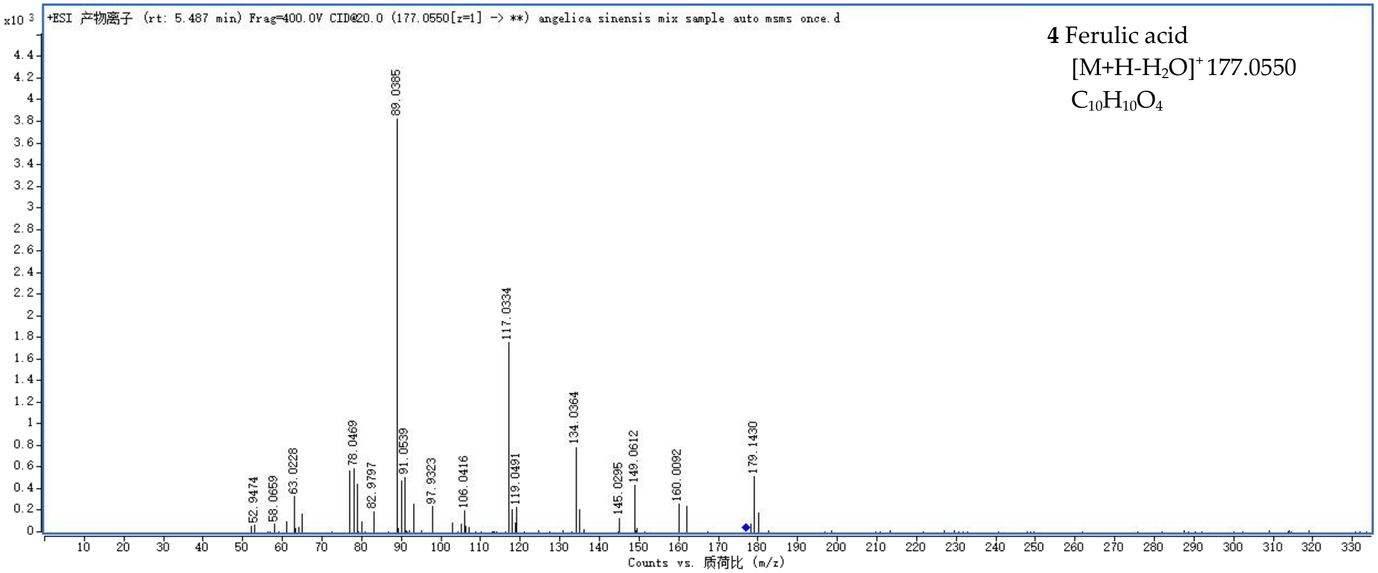
+ESI 产物离子 (rt: 5.487 min) Frag=400.0V CID@20.0 (177.0550[z=1] -> **) angelica sinensis mix sample auto msms once.d
x10 3
4 Ferulic acid
[M+H-H_2O]+ 177.0550
$C_{10}H_{10}O_4$
89.0385
117.0334
134.0364
179.1430
78.0469
91.0539
149.0612
160.0092
63.0228
82.9797
97.9323
106.0416
119.0491
145.0295
52.9474
58.0659
Counts vs. 质荷比 (m/z)

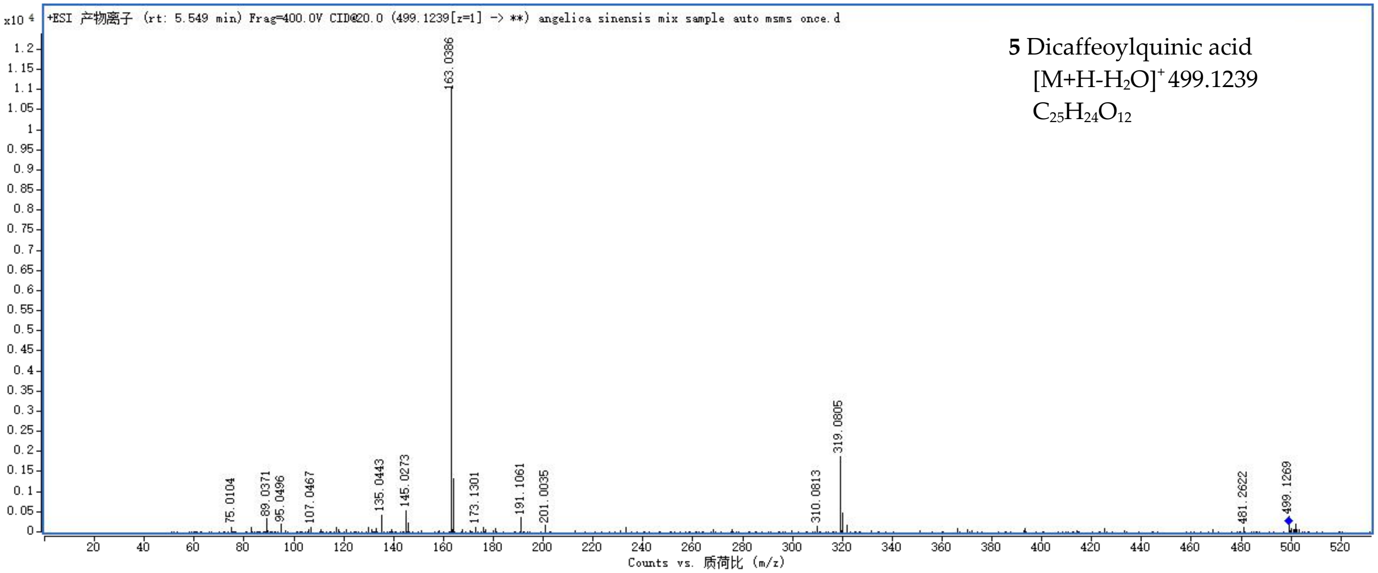

+ESI 产物离子 (rt: 5.549 min) Frag=400.0V CID@20.0 (499.1239[z=1] -> **) angelica sinensis mix sample auto msms once.d
5 Dicaffeoylquinic acid
[M+H-H2O]+ 499.1239
C25H24O12
163.0386
319.0805
75.0104
89.0371
95.0496
107.0467
135.0443
145.0273
173.1301
191.1061
201.0035
310.0813
481.2622
499.1269
Counts vs. 质荷比 (m/z)

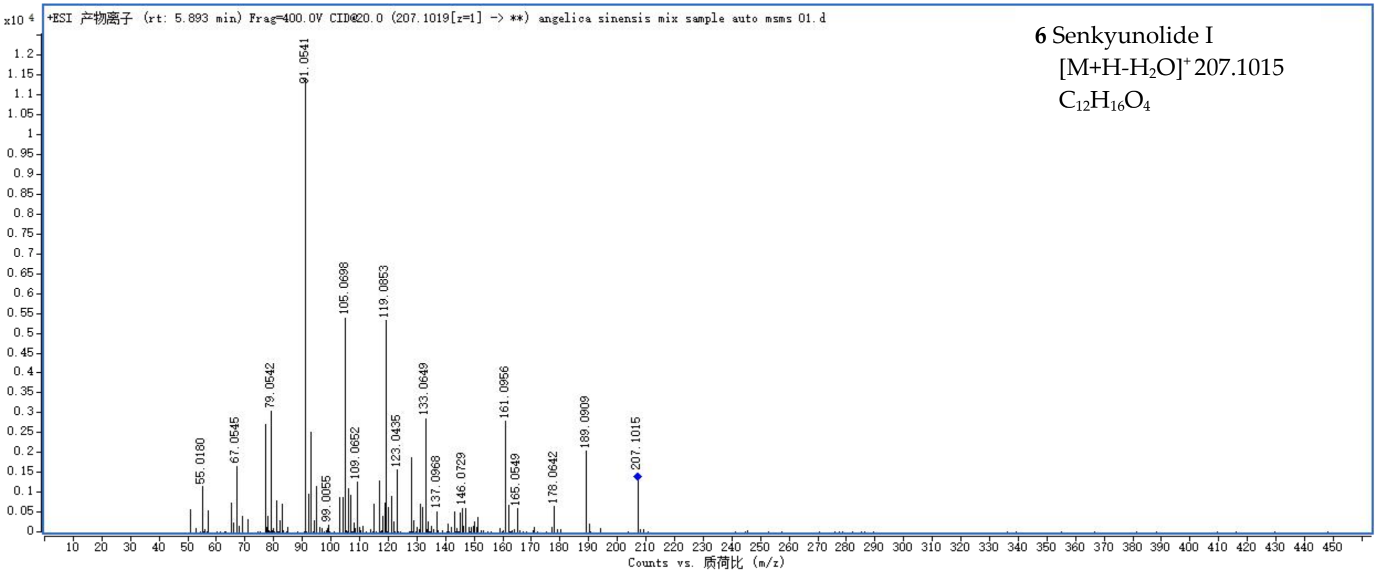

+ESI 产物离子 (rt: 5.893 min) Frag=400.0V CID@20.0 (207.1019[z=1] -> **) angelica sinensis mix sample auto msms 01.d
6 Senkyunolide I
[M+H-H2O]+ 207.1015
C12H16O4
91.0541
105.0698
119.0853
79.0542
133.0649
161.0956
189.0909
207.1015
55.0180
67.0545
99.0055
109.0652
123.0435
137.0968
146.0729
165.0549
178.0642
Counts vs. 质荷比 (m/z)

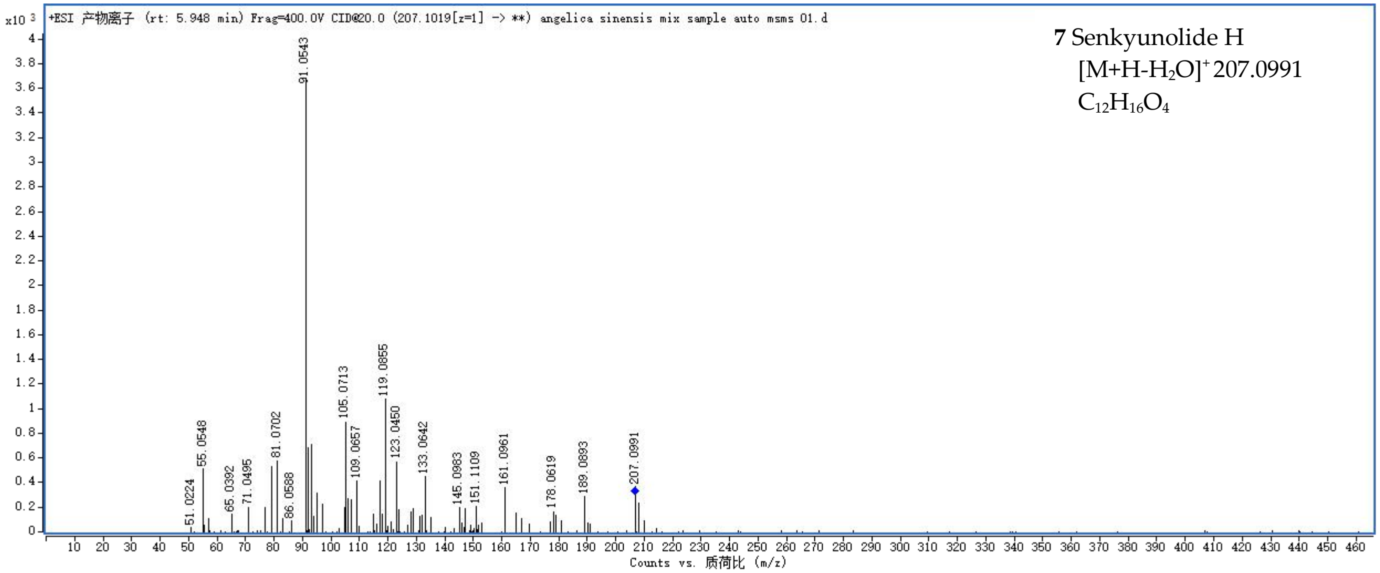
+ESI 产物离子 (rt: 5.948 min) Frag=400.0V CID@20.0 (207.1019[z=1] -> **) angelica sinensis mix sample auto msms 01.d
7 Senkyunolide H
$[M+H-H_2O]^+$ 207.0991
$C_{12}H_{16}O_4$
x10 3
51.0224
55.0548
65.0392
71.0495
81.0702
86.0588
91.0543
105.0713
109.0657
119.0855
123.0450
133.0642
145.0983
151.1109
161.0961
178.0619
189.0893
207.0991
Counts vs. 质荷比 (m/z)

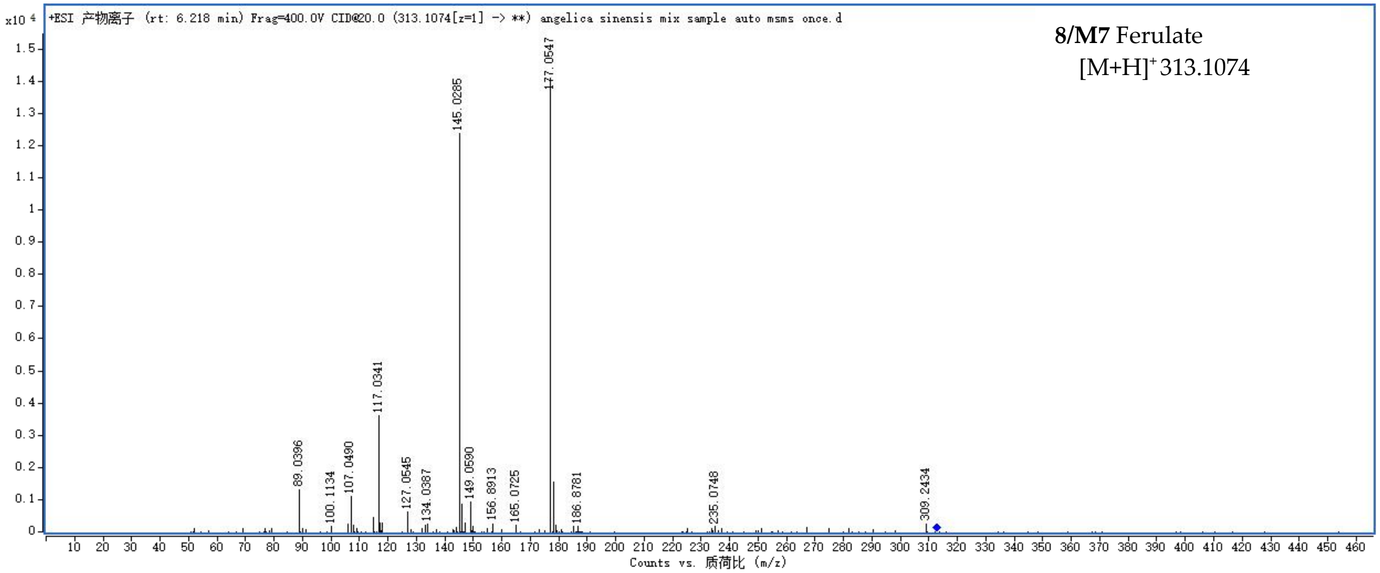
+ESI 产物离子 (rt: 6.218 min) Frag=400.0V CID@20.0 (313.1074[z=1] -> **) angelica sinensis mix sample auto msms once.d
8/M7 Ferulate
$[M+H]^+$ 313.1074
x10 4
89.0396
100.1134
107.0490
117.0341
127.0545
134.0387
145.0285
149.0590
156.8913
165.0725
177.0547
186.8781
235.0748
309.2434
Counts vs. 质荷比 (m/z)

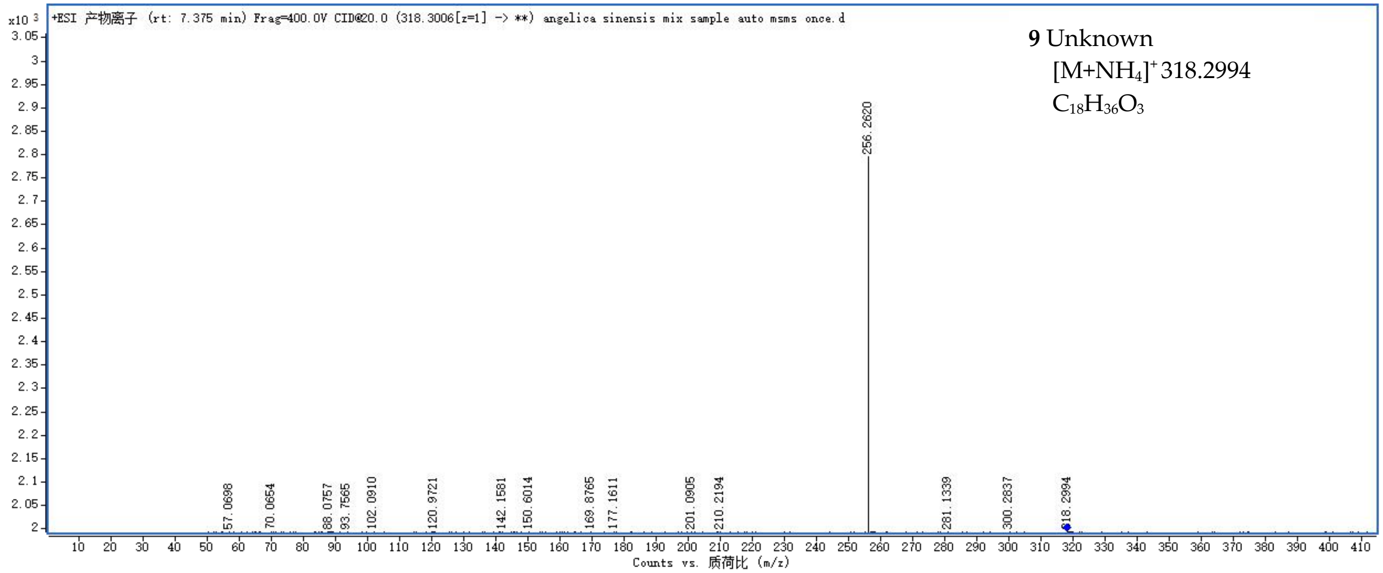
+ESI 产物离子 (rt: 7.375 min) Frag=400.0V CID@20.0 (318.3006[z=1] -> **) angelica sinensis mix sample auto msms once.d
9 Unknown
[M+NH4]+ 318.2994
C18H36O3
256.2620
Counts vs. 质荷比 (m/z)

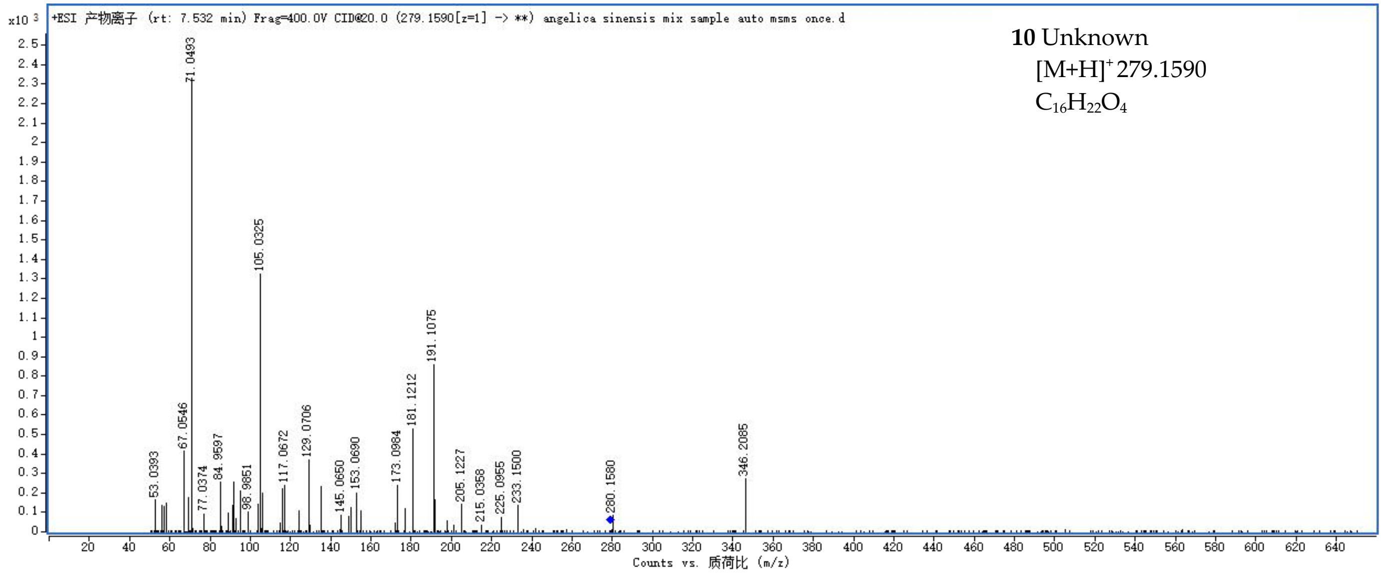
+ESI 产物离子 (rt: 7.532 min) Frag=400.0V CID@20.0 (279.1590[z=1] -> **) angelica sinensis mix sample auto msms once.d
10 Unknown
[M+H]+ 279.1590
C16H22O4
71.0493
105.0325
191.1075
346.2085
Counts vs. 质荷比 (m/z)

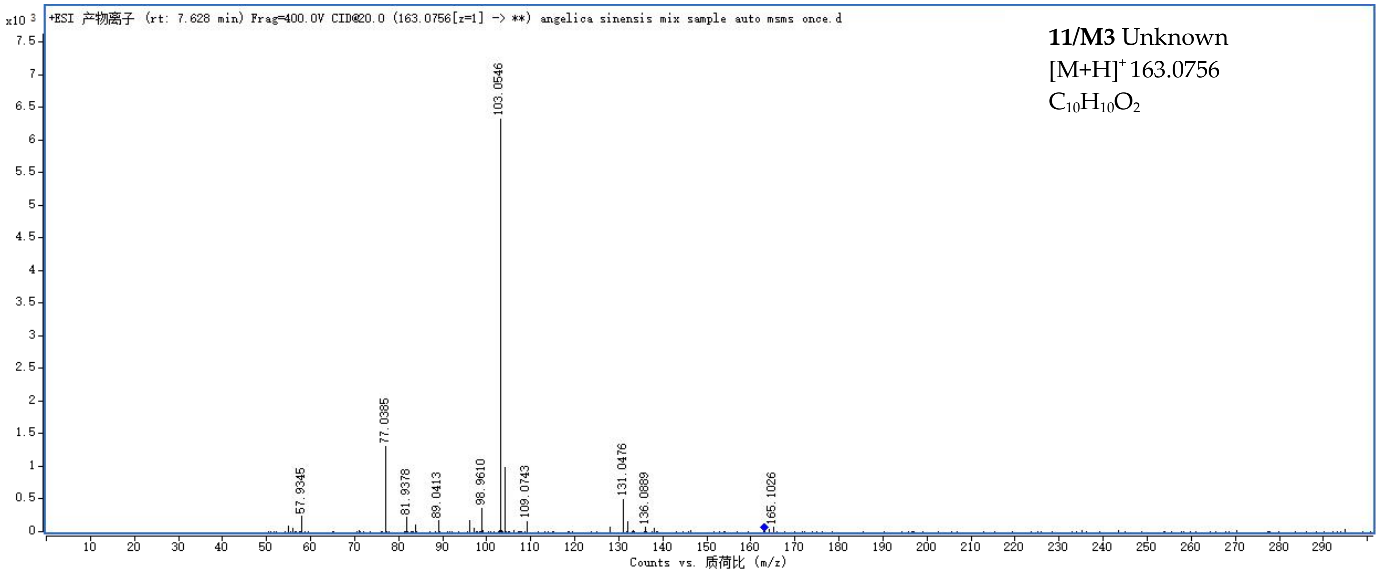
+ESI 产物离子 (rt: 7.628 min) Frag=400.0V CID@20.0 (163.0756[z=1] -> **) angelica sinensis mix sample auto msms once.d
11/M3 Unknown
$[M+H]^+$ 163.0756
$C_{10}H_{10}O_2$
x10 3
103.0546
77.0385
57.9345
81.9378
89.0413
98.9610
109.0743
131.0476
136.0889
165.1026
Counts vs. 质荷比 (m/z)

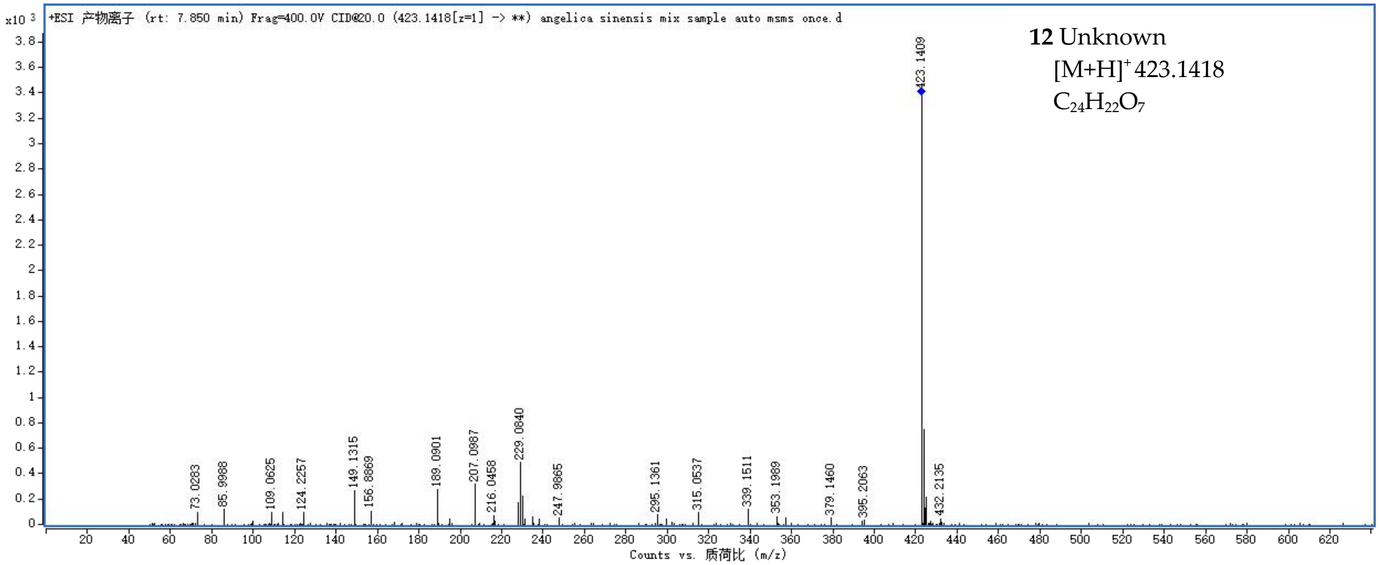
+ESI 产物离子 (rt: 7.850 min) Frag=400.0V CID@20.0 (423.1418[z=1] -> **) angelica sinensis mix sample auto msms once.d
12 Unknown
$[M+H]^+$ 423.1418
$C_{24}H_{22}O_7$
x10 3
423.1409
73.0283
85.9988
109.0625
124.2257
149.1315
156.8869
189.0901
207.0987
216.0458
229.0840
247.9865
295.1361
315.0537
339.1511
353.1989
379.1460
395.2063
432.2135
Counts vs. 质荷比 (m/z)

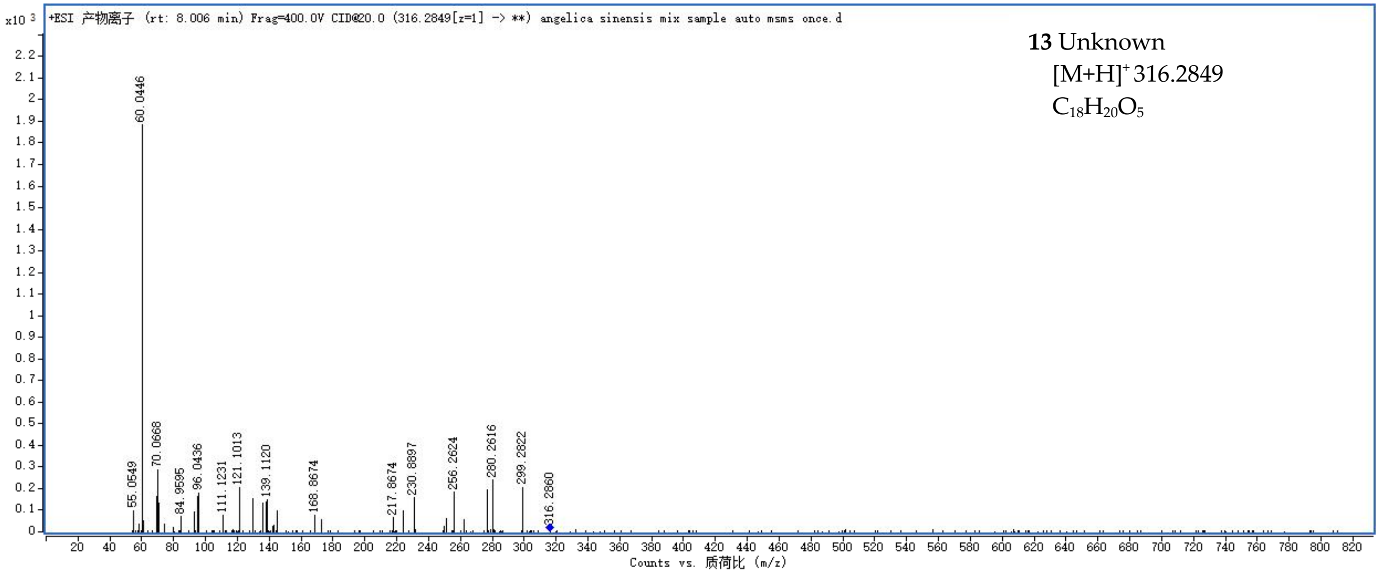
+ESI 产物离子 (rt: 8.006 min) Frag=400.0V CID@20.0 (316.2849[z=1] -> **) angelica sinensis mix sample auto msms once.d
13 Unknown
[M+H]+ 316.2849
C18H20O5
60.0446
55.0549
70.0668
84.9595
96.0436
111.1231
121.1013
139.1120
168.8674
217.8674
230.8897
256.2624
280.2616
299.2822
316.2860
Counts vs. 质荷比 (m/z)

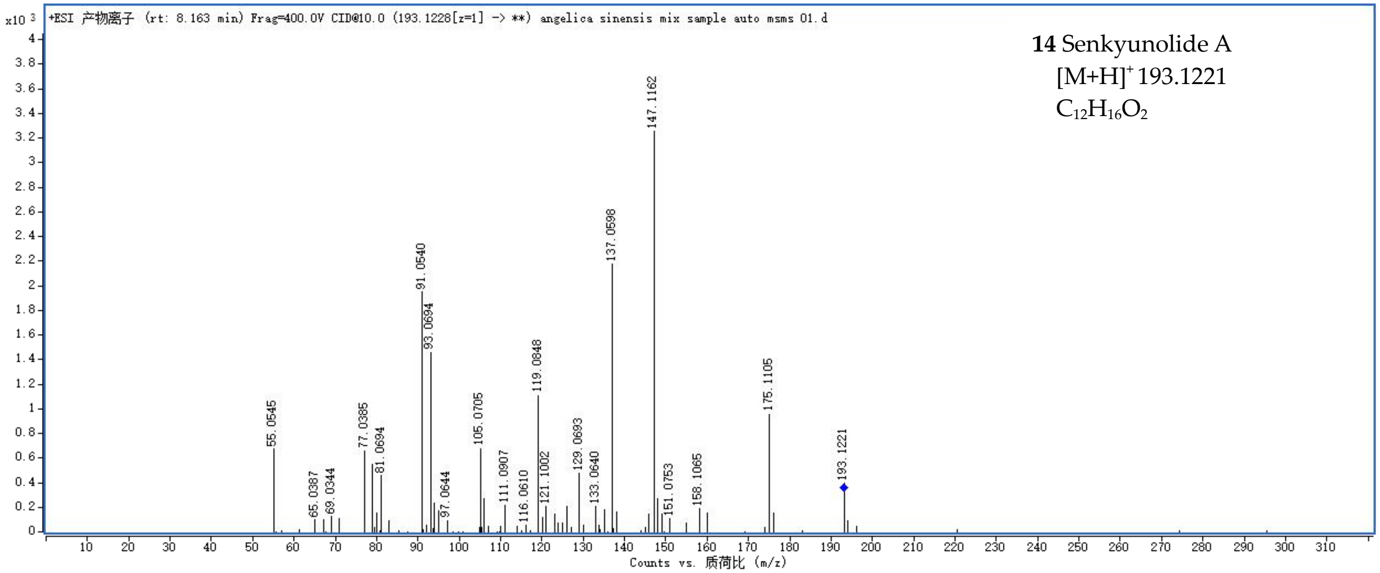
+ESI 产物离子 (rt: 8.163 min) Frag=400.0V CID@10.0 (193.1228[z=1] -> **) angelica sinensis mix sample auto msms 01.d
14 Senkyunolide A
[M+H]+ 193.1221
C12H16O2
147.1162
137.0598
91.0540
93.0694
119.0848
175.1105
105.0705
77.0385
55.0545
81.0694
129.0693
133.0640
111.0907
121.1002
116.0610
65.0387
69.0344
97.0644
151.0753
158.1065
193.1221
Counts vs. 质荷比 (m/z)

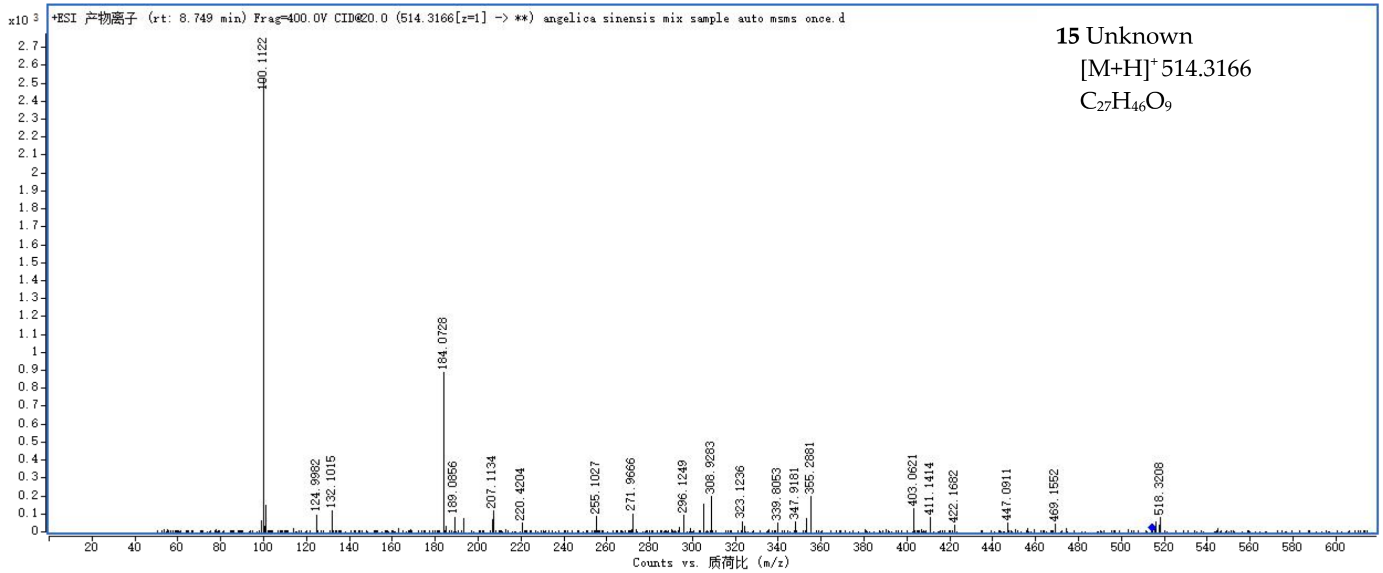
+ESI 产物离子 (rt: 8.749 min) Frag=400.0V CID@20.0 (514.3166[z=1] -> **) angelica sinensis mix sample auto msms once.d
15 Unknown
$[M+H]^+$ 514.3166
$C_{27}H_{46}O_9$
x10 3
100.1122
124.9982
132.1015
184.0728
189.0856
207.1134
220.4204
255.1027
271.9666
296.1249
308.9283
323.1236
339.8053
347.9181
355.2881
403.0621
411.1414
422.1682
447.0911
469.1552
518.3208
Counts vs. 质荷比 (m/z)

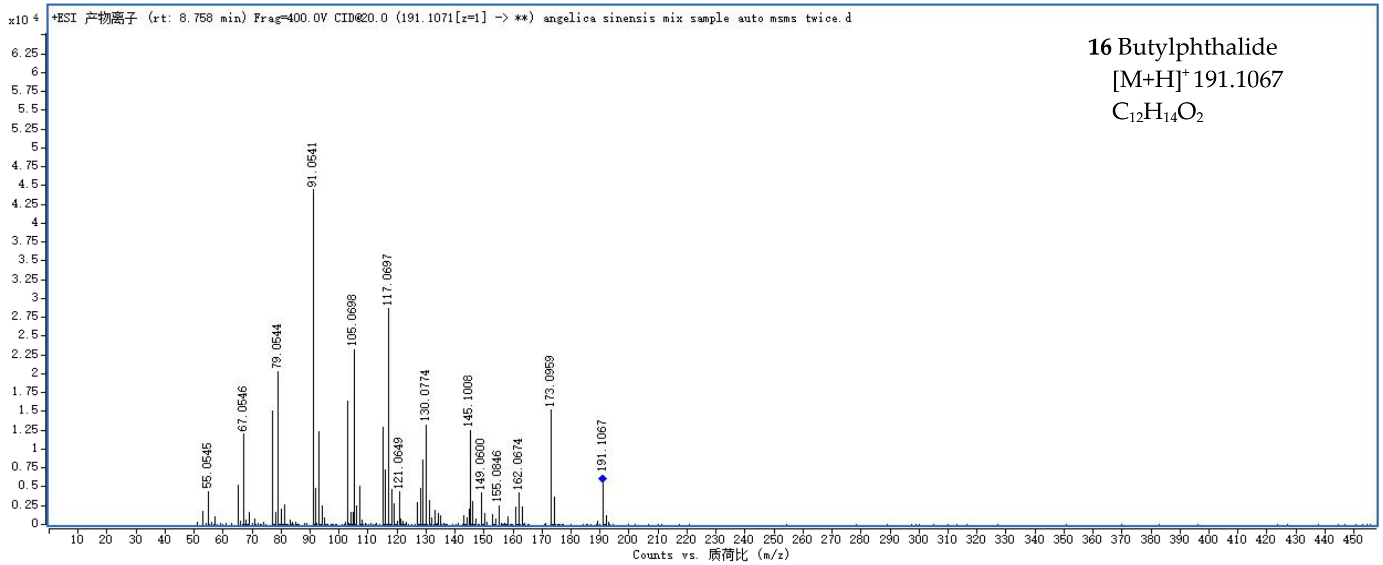
+ESI 产物离子 (rt: 8.758 min) Frag=400.0V CID@20.0 (191.1071[z=1] -> **) angelica sinensis mix sample auto msms twice.d
16 Butylphthalide
$[M+H]^+$ 191.1067
$C_{12}H_{14}O_2$
x10 4
55.0545
67.0546
79.0544
91.0541
105.0698
117.0697
121.0649
130.0774
145.1008
149.0600
155.0846
162.0674
173.0959
191.1067
Counts vs. 质荷比 (m/z)

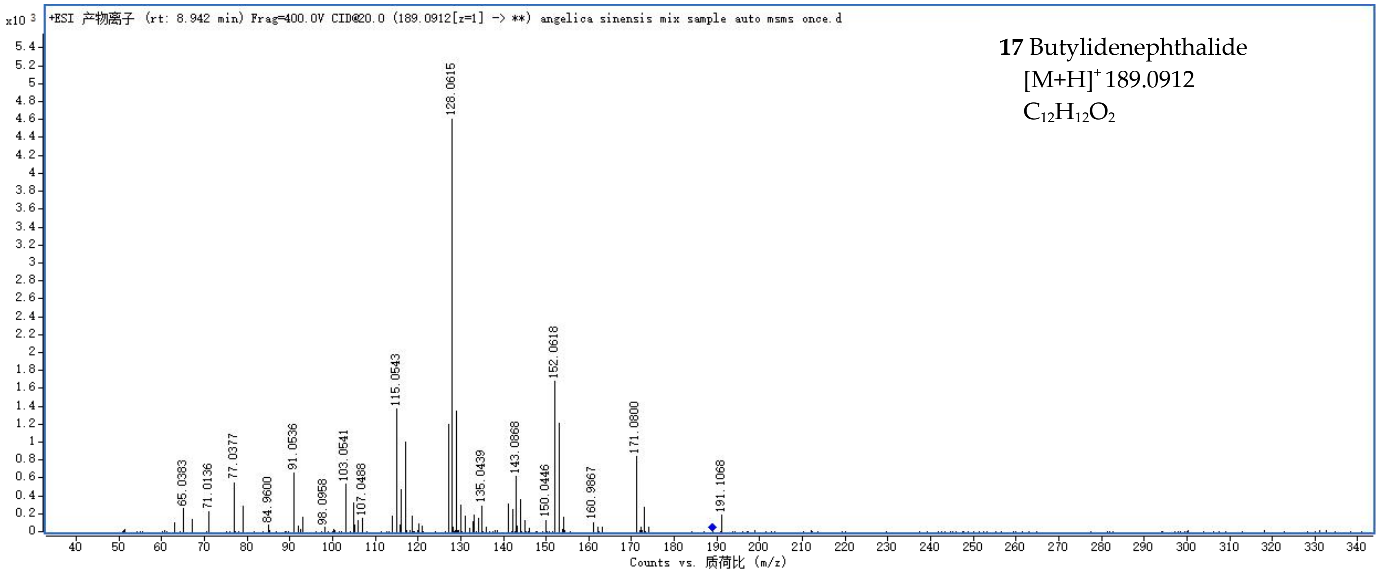
+ESI 产物离子 (rt: 8.942 min) Frag=400.0V CID@20.0 (189.0912[z=1] -> **) angelica sinensis mix sample auto msms once.d
x10 3
17 Butylidenephthalide
[M+H]+ 189.0912
C12H12O2
65.0383
71.0136
77.0377
84.9600
91.0536
98.0958
103.0541
107.0488
115.0543
128.0615
135.0439
143.0868
150.0446
152.0618
160.9867
171.0800
191.1068
Counts vs. 质荷比 (m/z)

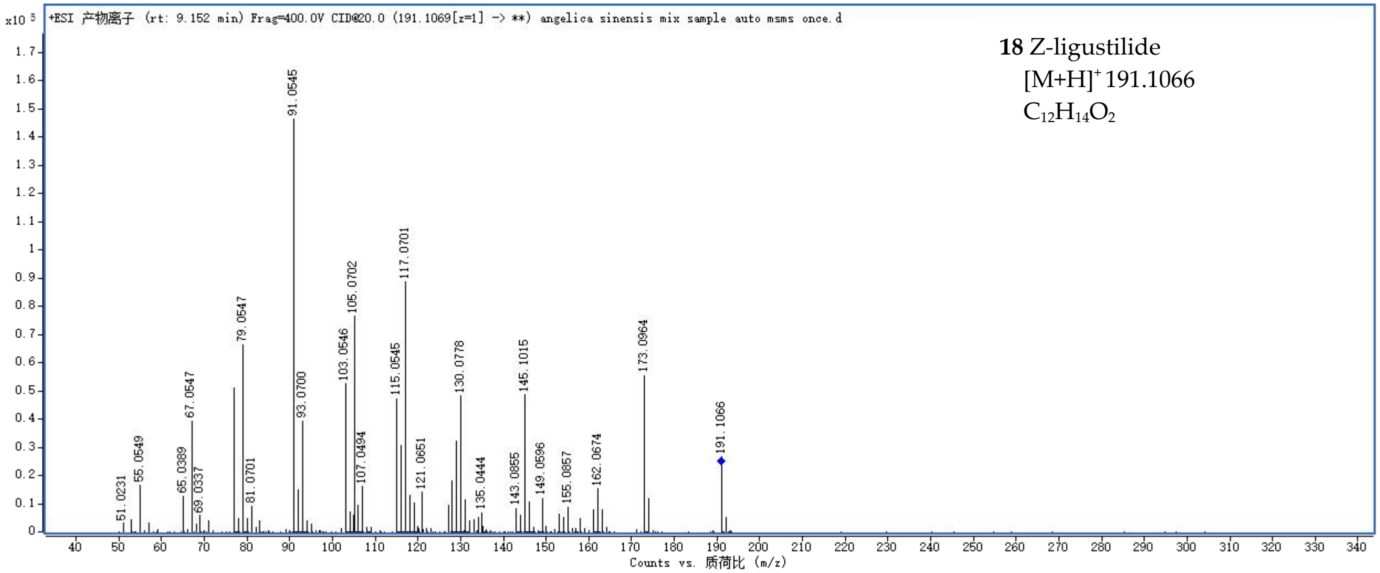
+ESI 产物离子 (rt: 9.152 min) Frag=400.0V CID@20.0 (191.1069[z=1] -> **) angelica sinensis mix sample auto msms once.d
x10 5
18 Z-ligustilide
[M+H]+ 191.1066
C12H14O2
51.0231
55.0549
65.0389
67.0547
69.0337
79.0547
81.0701
91.0545
93.0700
103.0546
105.0702
107.0494
115.0545
117.0701
121.0651
130.0778
135.0444
143.0855
145.1015
149.0596
155.0857
162.0674
173.0964
191.1066
Counts vs. 质荷比 (m/z)

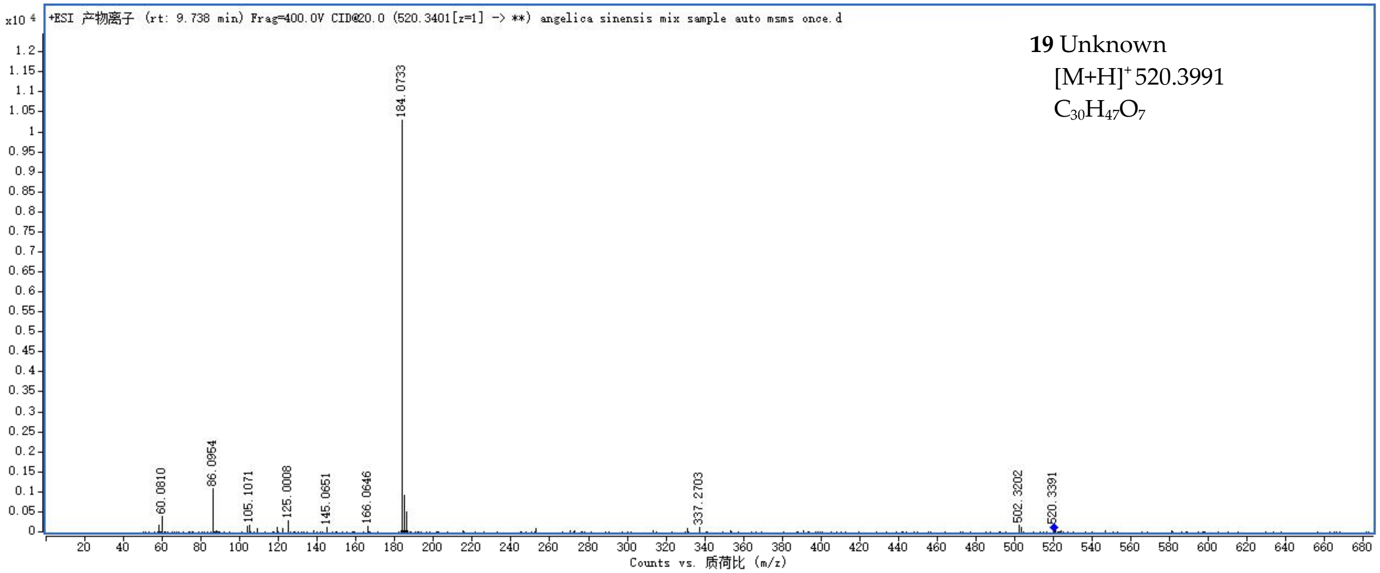

x10 4
+ESI 产物离子 (rt: 9.738 min) Frag=400.0V CID@20.0 (520.3401[z=1] -> **) angelica sinensis mix sample auto msms once.d
19 Unknown
[M+H]+ 520.3991
C30H47O7
184.0733
60.0810
86.0954
105.1071
125.0008
145.0651
166.0646
337.2703
502.3202
520.3391
Counts vs. 质荷比 (m/z)

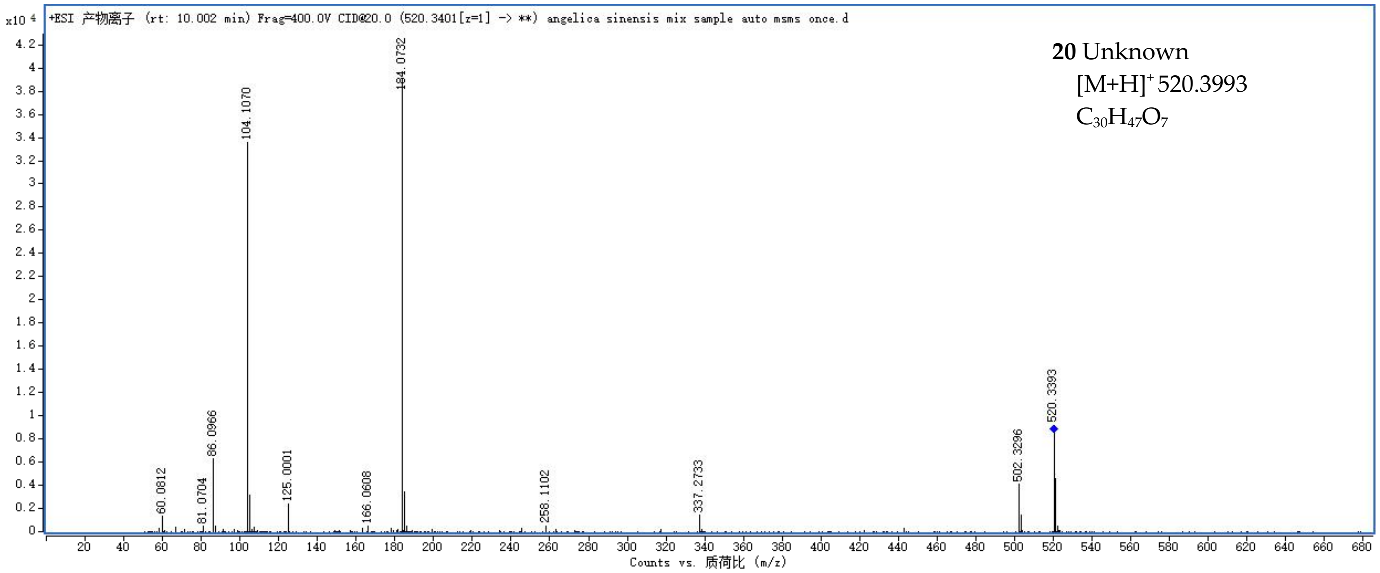

x10 4
+ESI 产物离子 (rt: 10.002 min) Frag=400.0V CID@20.0 (520.3401[z=1] -> **) angelica sinensis mix sample auto msms once.d
20 Unknown
[M+H]+ 520.3993
C30H47O7
184.0732
104.1070
60.0812
81.0704
86.0966
125.0001
166.0608
258.1102
337.2733
502.3296
520.3393
Counts vs. 质荷比 (m/z)

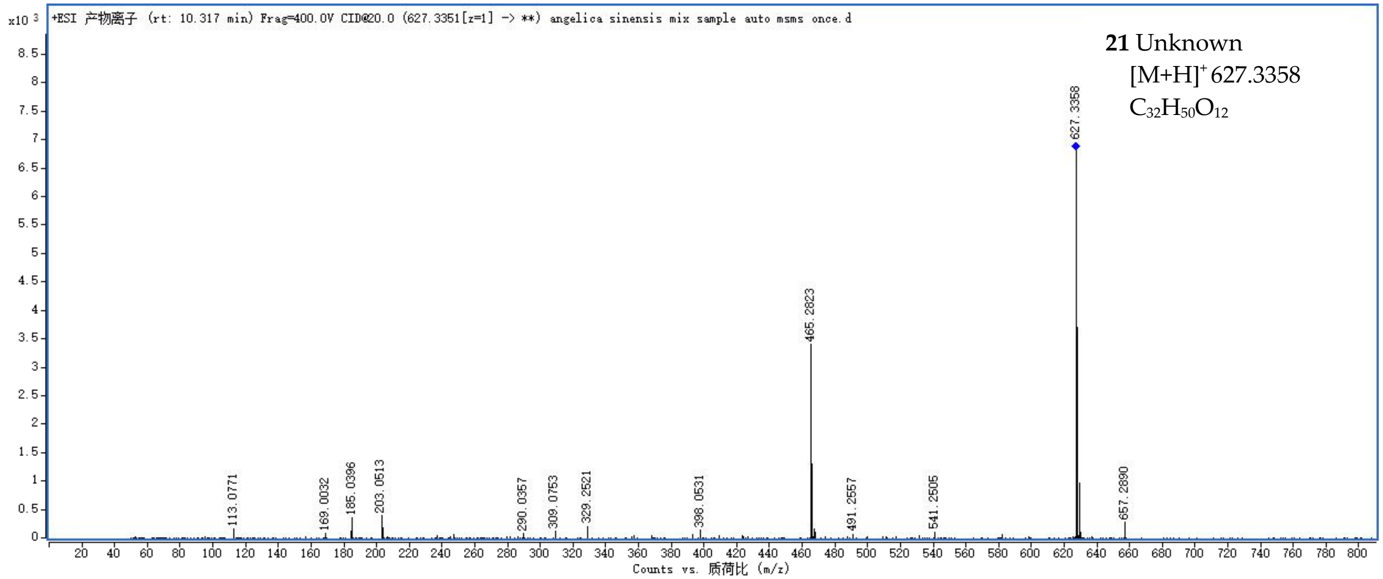
+ESI 产物离子 (rt: 10.317 min) Frag=400.0V CID@20.0 (627.3351[z=1] -> **) angelica sinensis mix sample auto msms once.d
21 Unknown
[M+H]+ 627.3358
C32H50O12
627.3358
465.2823
113.0771
169.0032
185.0396
203.0513
290.0357
309.0753
329.2521
398.0531
491.2557
541.2505
657.2890
x10 3
Counts vs. 质荷比 (m/z)

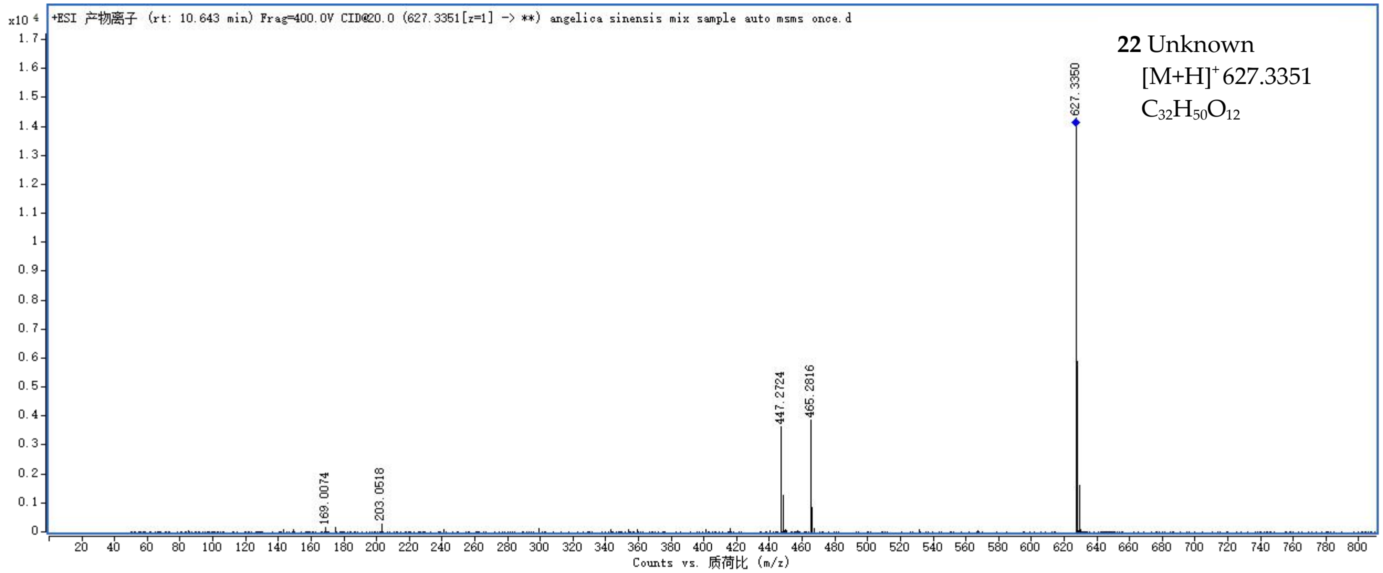
+ESI 产物离子 (rt: 10.643 min) Frag=400.0V CID@20.0 (627.3351[z=1] -> **) angelica sinensis mix sample auto msms once.d
22 Unknown
[M+H]+ 627.3351
C32H50O12
627.3350
447.2724
465.2816
169.0074
203.0518
x10 4
Counts vs. 质荷比 (m/z)

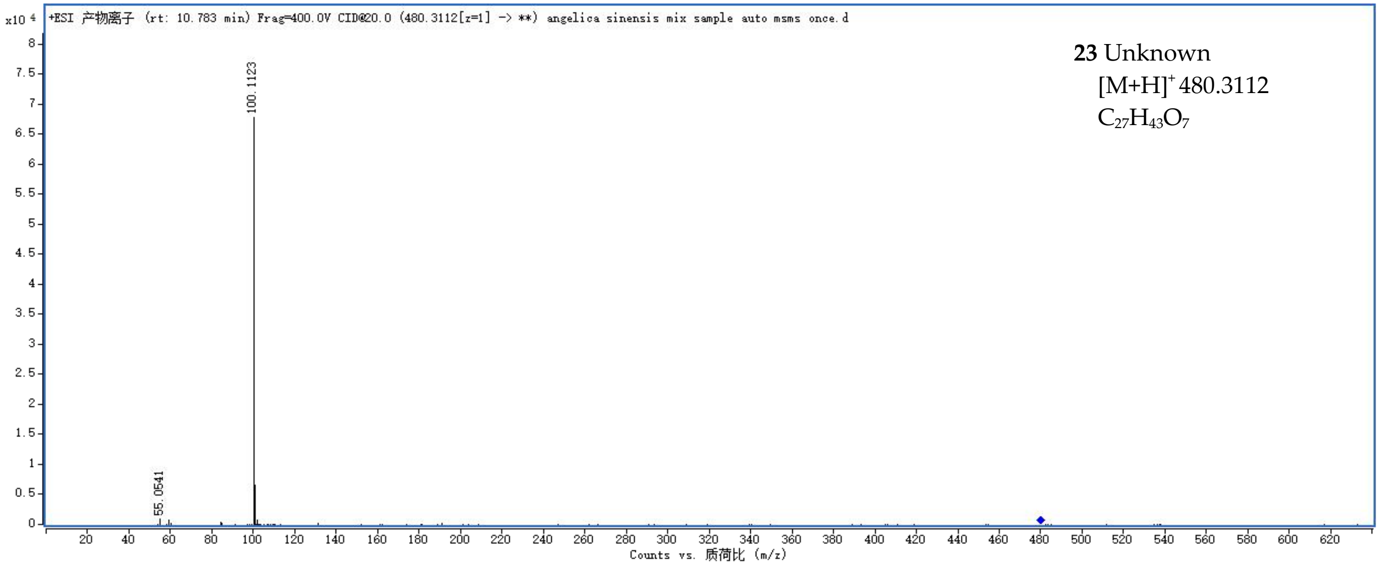
x10 4
+ESI 产物离子 (rt: 10.783 min) Frag=400.0V CID@20.0 (480.3112[z=1] -> **) angelica sinensis mix sample auto msms once.d
23 Unknown
[M+H]+ 480.3112
C27H43O7
100.1123
55.0541
Counts vs. 质荷比 (m/z)

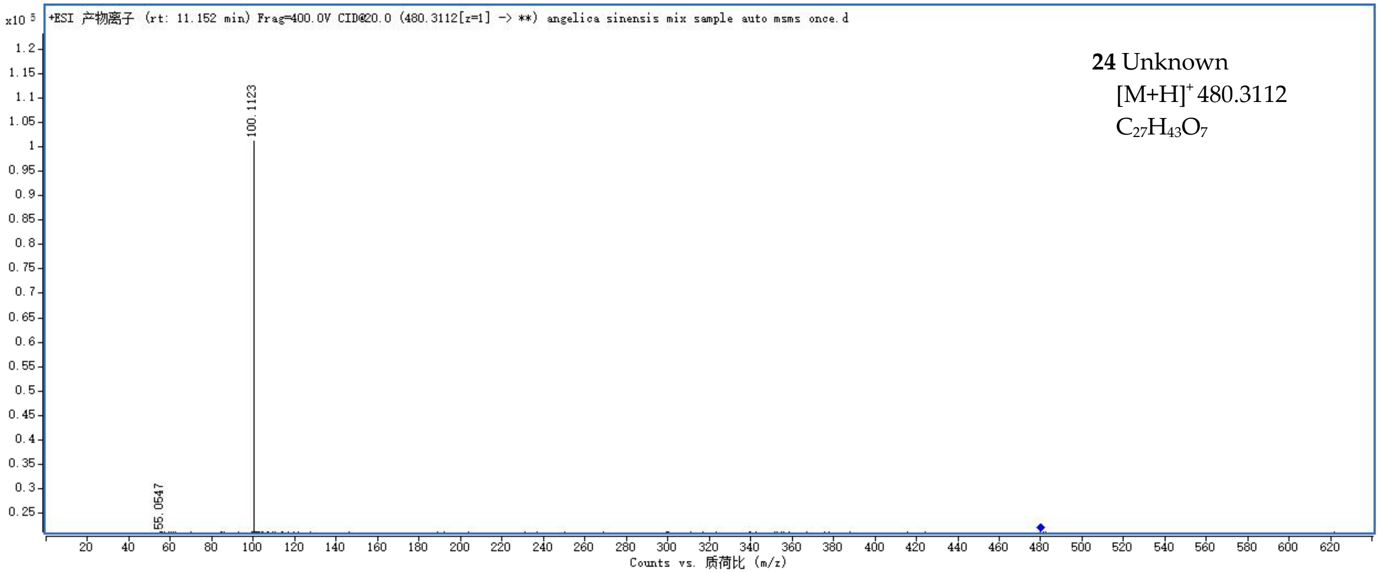
x10 5
+ESI 产物离子 (rt: 11.152 min) Frag=400.0V CID@20.0 (480.3112[z=1] -> **) angelica sinensis mix sample auto msms once.d
24 Unknown
[M+H]+ 480.3112
C27H43O7
100.1123
55.0547
Counts vs. 质荷比 (m/z)

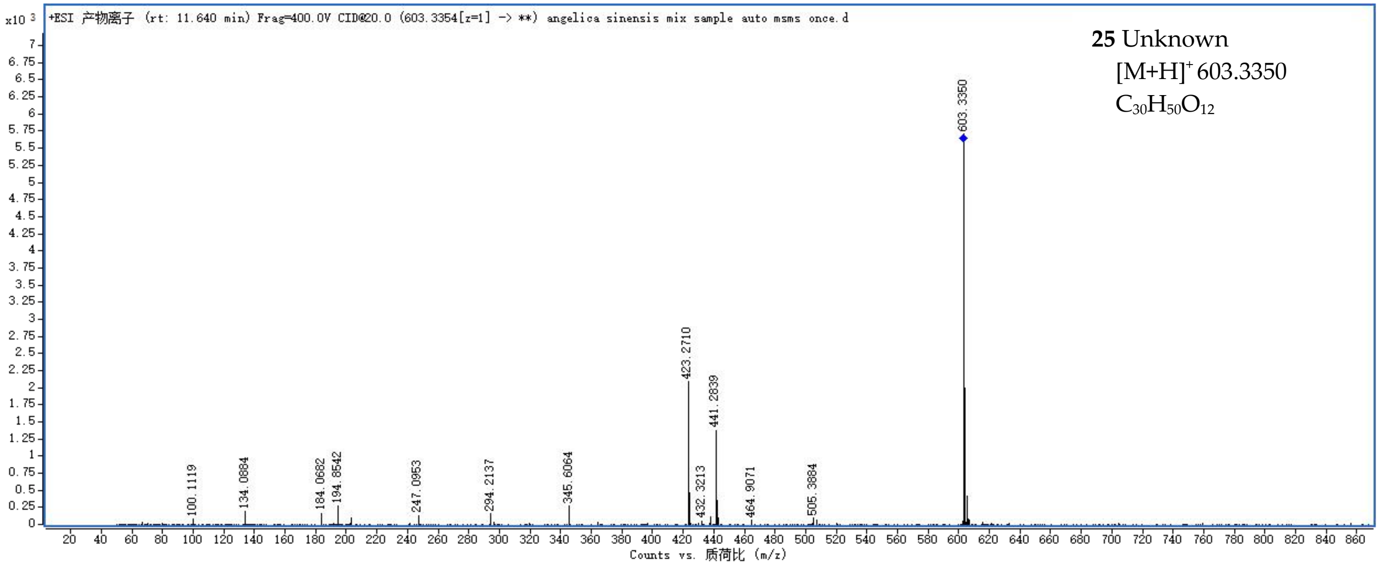
+ESI 产物离子 (rt: 11.640 min) Frag=400.0V CID@20.0 (603.3354[z=1] -> **) angelica sinensis mix sample auto msms once.d
x10 3
25 Unknown
[M+H]+ 603.3350
$C_{30}H_{50}O_{12}$
603.3350
423.2710
441.2839
432.3213
464.9071
505.3884
345.6064
294.2137
247.0953
184.0682
194.8542
134.0884
100.1119
Counts vs. 质荷比 (m/z)

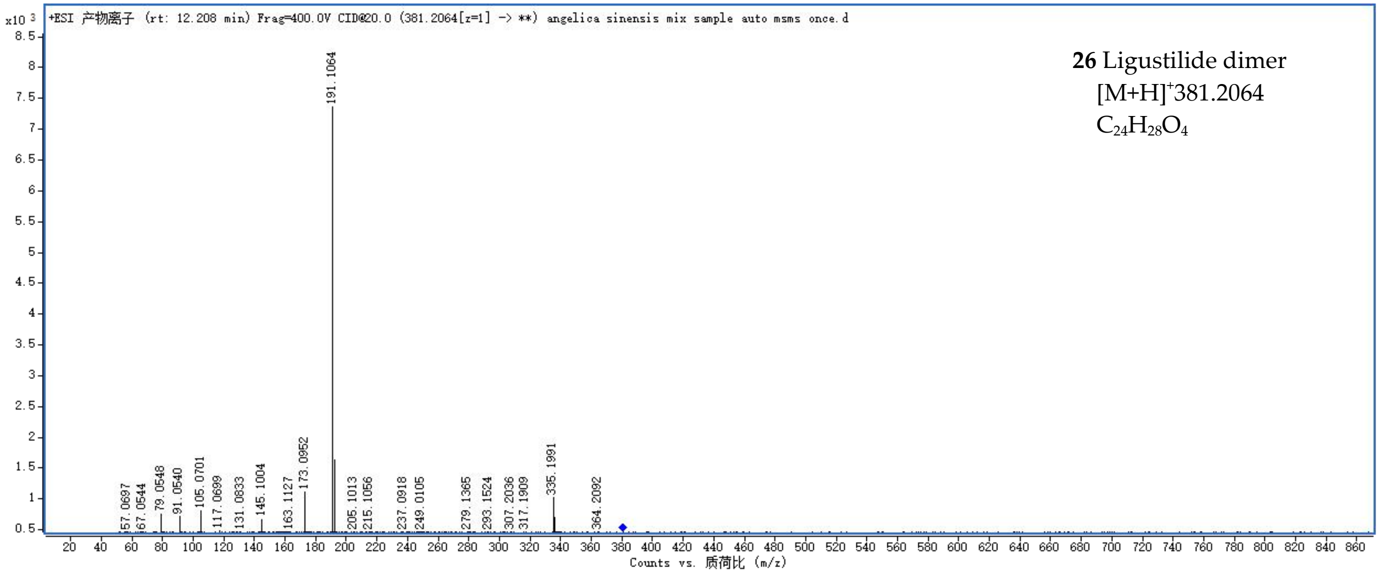
+ESI 产物离子 (rt: 12.208 min) Frag=400.0V CID@20.0 (381.2064[z=1] -> **) angelica sinensis mix sample auto msms once.d
x10 3
26 Ligustilide dimer
[M+H]+381.2064
$C_{24}H_{28}O_4$
191.1064
173.0952
335.1991
57.0697
67.0544
79.0548
91.0540
105.0701
117.0699
131.0833
145.1004
163.1127
205.1013
215.1056
237.0918
249.0105
279.1365
293.1524
307.2036
317.1909
364.2092
Counts vs. 质荷比 (m/z)

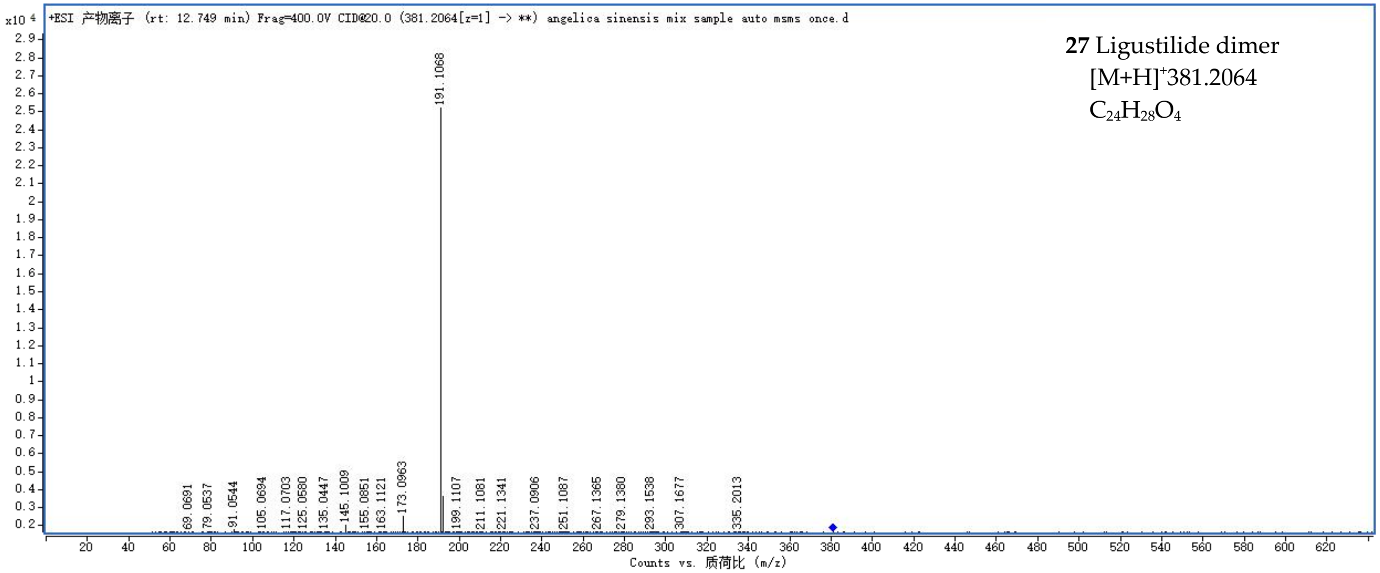
+ESI 产物离子 (rt: 12.749 min) Frag=400.0V CID@20.0 (381.2064[z=1] -> **) angelica sinensis mix sample auto msms once.d
27 Ligustilide dimer
[M+H]+381.2064
$C_{24}H_{28}O_4$
191.1068
69.0691 79.0537 91.0544 105.0694 117.0703 125.0580 135.0447 145.1009 155.0851 163.1121 173.0963 199.1107 211.1081 221.1341 237.0906 251.1087 267.1365 279.1380 293.1538 307.1677 335.2013
x10 4
Counts vs. 质荷比 (m/z)

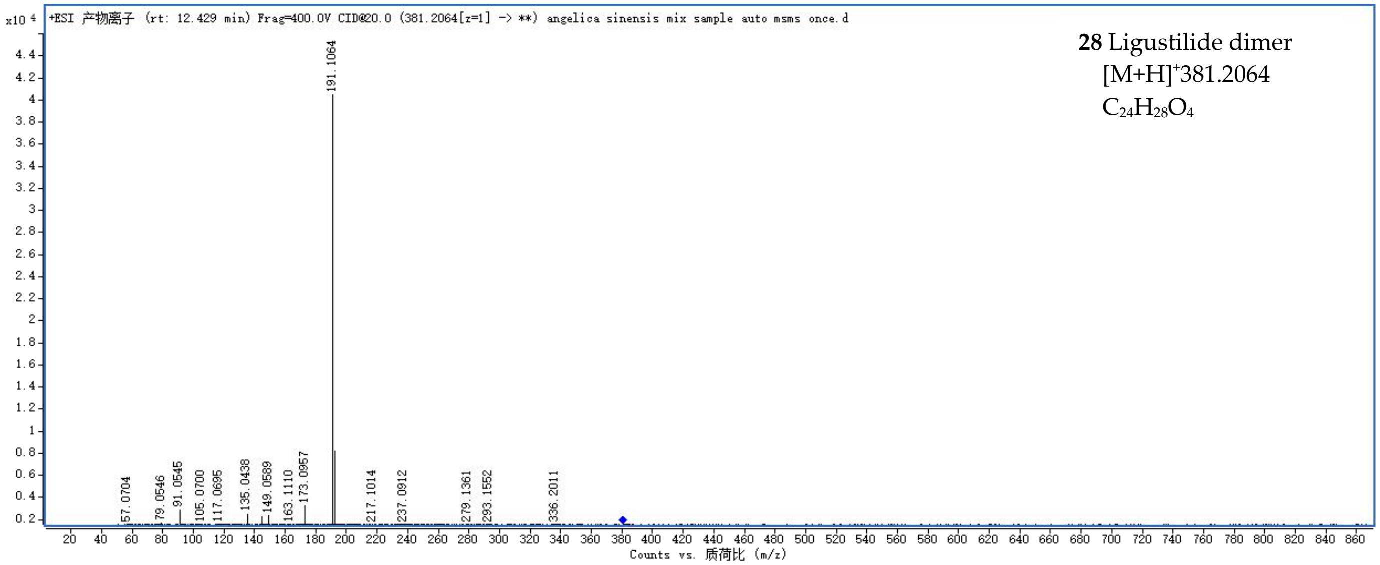
+ESI 产物离子 (rt: 12.429 min) Frag=400.0V CID@20.0 (381.2064[z=1] -> **) angelica sinensis mix sample auto msms once.d
28 Ligustilide dimer
[M+H]+381.2064
$C_{24}H_{28}O_4$
191.1064
57.0704 79.0546 91.0545 105.0700 117.0695 135.0438 149.0589 163.1110 173.0957 217.1014 237.0912 279.1361 293.1552 336.2011
x10 4
Counts vs. 质荷比 (m/z)

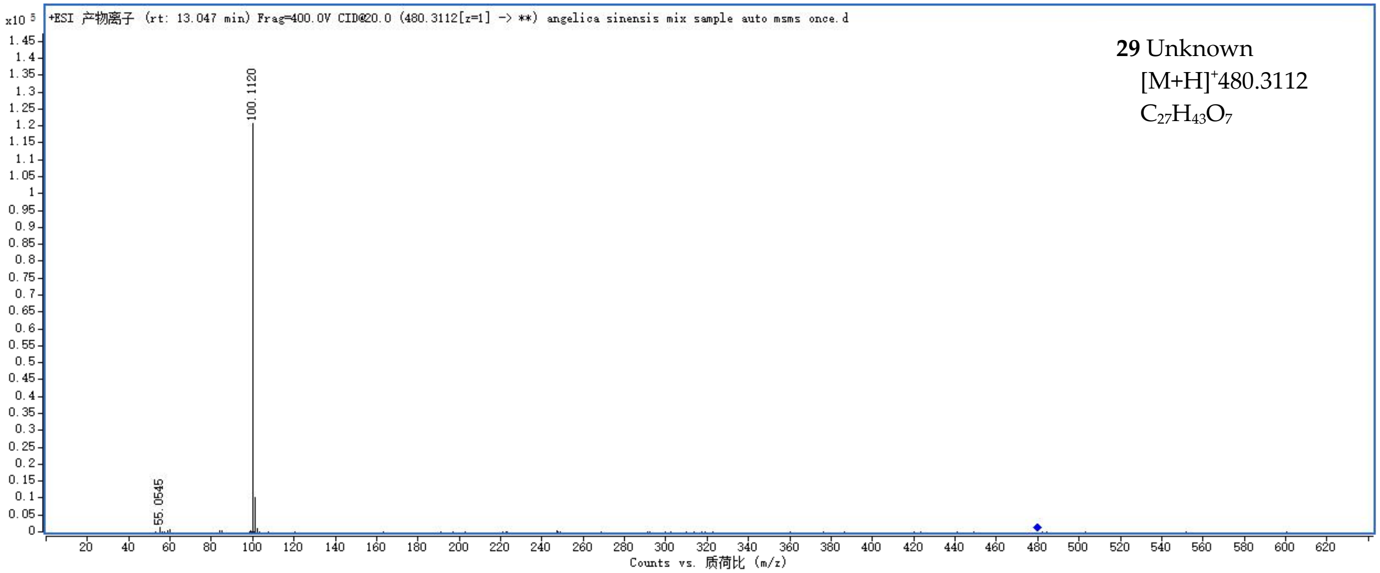

+ESI 产物离子 (rt: 13.047 min) Frag=400.0V CID@20.0 (480.3112[z=1] -> **) angelica sinensis mix sample auto msms once.d
x10 5
100.1120
55.0545
Counts vs. 质荷比 (m/z)
29 Unknown
[M+H]+480.3112
C27H43O7

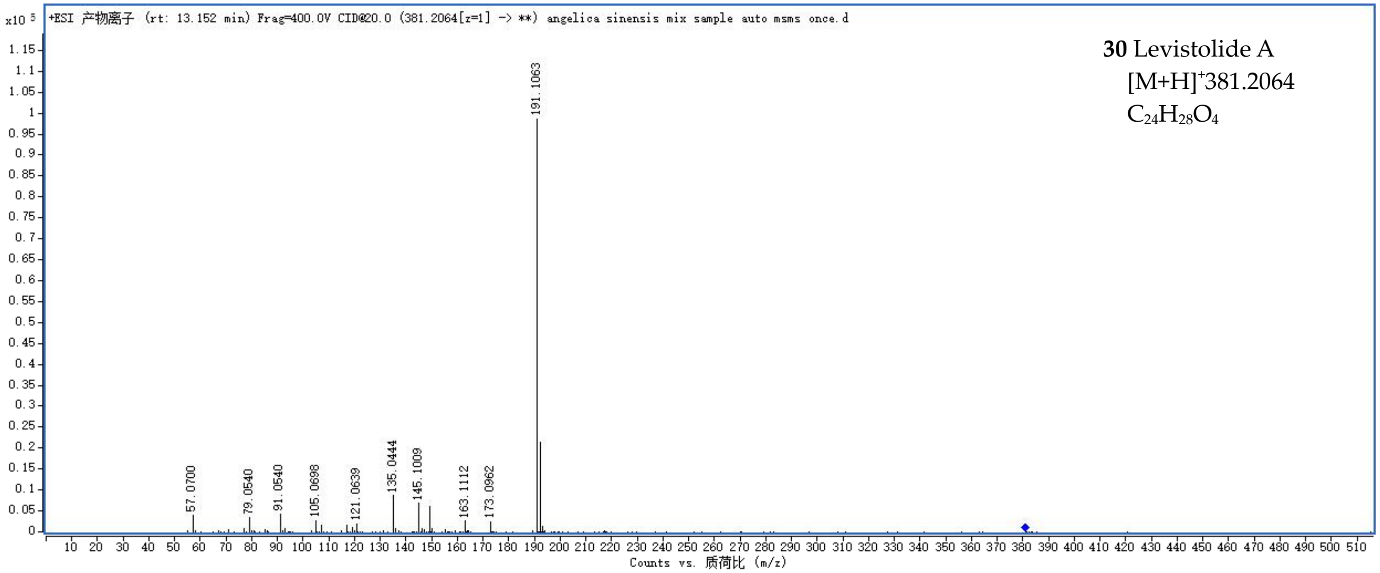

+ESI 产物离子 (rt: 13.152 min) Frag=400.0V CID@20.0 (381.2064[z=1] -> **) angelica sinensis mix sample auto msms once.d
x10 5
191.1063
57.0700
79.0540
91.0540
105.0698
121.0639
135.0444
145.1009
163.1112
173.0962
Counts vs. 质荷比 (m/z)
30 Levistolide A
[M+H]+381.2064
C24H28O4

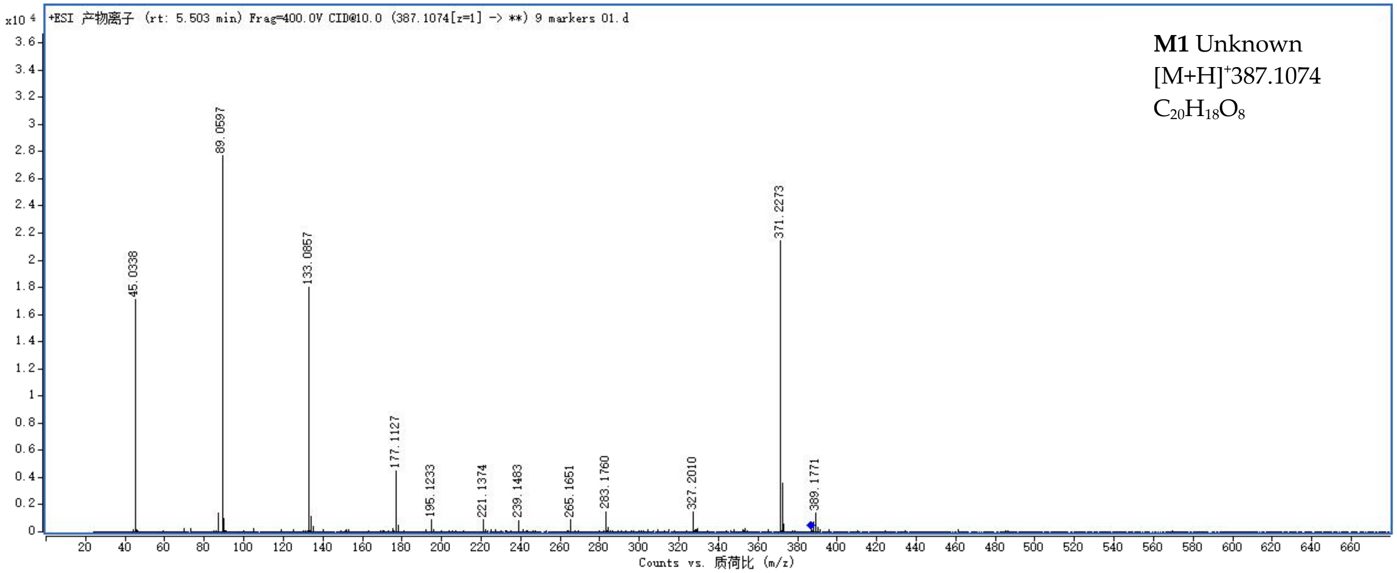
+ESI 产物离子 (rt: 5.503 min) Frag=400.0V CID@10.0 (387.1074[z=1] -> **) 9 markers 01.d
M1 Unknown
[M+H]+387.1074
C20H18O8
Counts vs. 质荷比 (m/z)

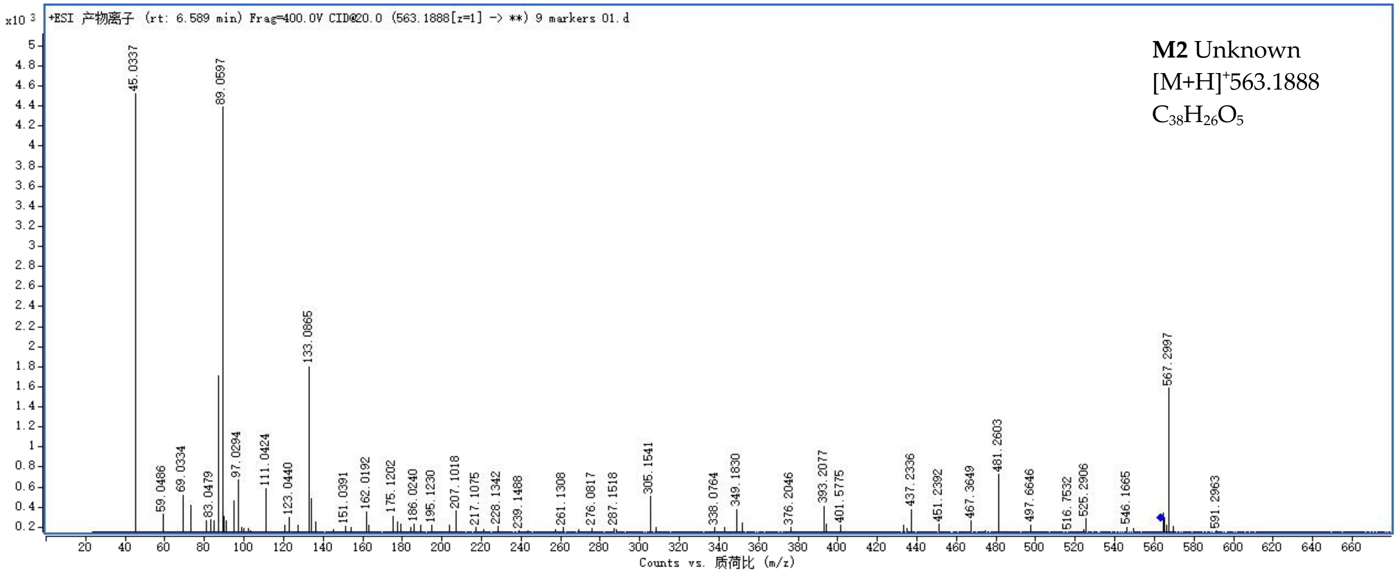
+ESI 产物离子 (rt: 6.589 min) Frag=400.0V CID@20.0 (563.1888[z=1] -> **) 9 markers 01.d
M2 Unknown
[M+H]+563.1888
C38H26O5
Counts vs. 质荷比 (m/z)

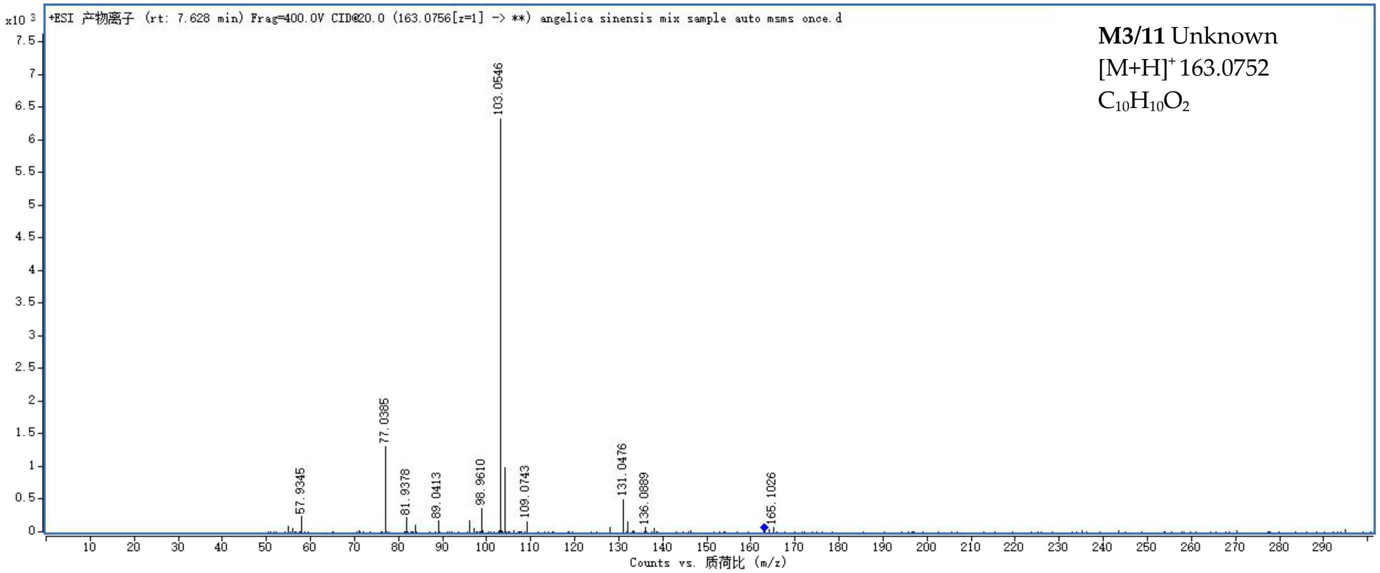
+ESI 产物离子 (rt: 7.628 min) Frag=400.0V CID@20.0 (163.0756[z=1] -> **) angelica sinensis mix sample auto msms once.d
M3/11 Unknown
[M+H]+ 163.0752
$C_{10}H_{10}O_2$
x10 3
103.0546
77.0385
57.9345
81.9378
89.0413
98.9610
109.0743
131.0476
136.0889
165.1026
Counts vs. 质荷比 (m/z)

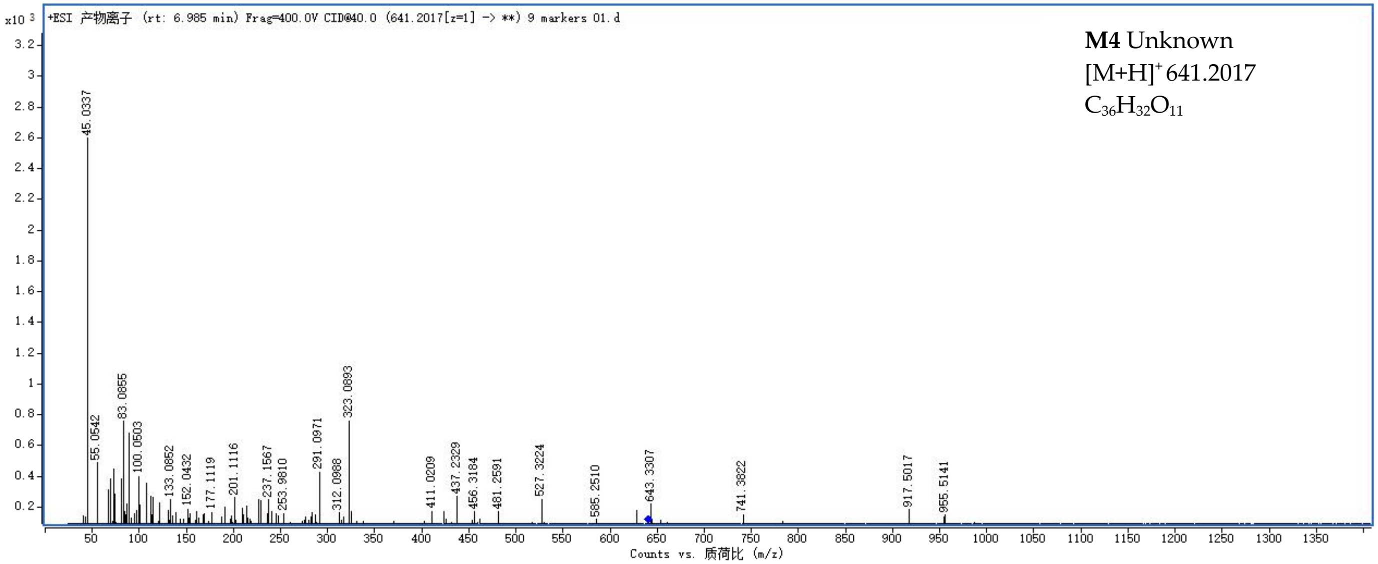
+ESI 产物离子 (rt: 6.985 min) Frag=400.0V CID@40.0 (641.2017[z=1] -> **) 9 markers 01.d
M4 Unknown
[M+H]+ 641.2017
$C_{36}H_{32}O_{11}$
x10 3
45.0337
55.0542
83.0855
100.0503
133.0852
152.0432
177.1119
201.1116
237.1567
253.9810
291.0971
312.0988
323.0893
411.0209
437.2329
456.3184
481.2591
527.3224
585.2510
643.3307
741.3822
917.5017
955.5141
Counts vs. 质荷比 (m/z)

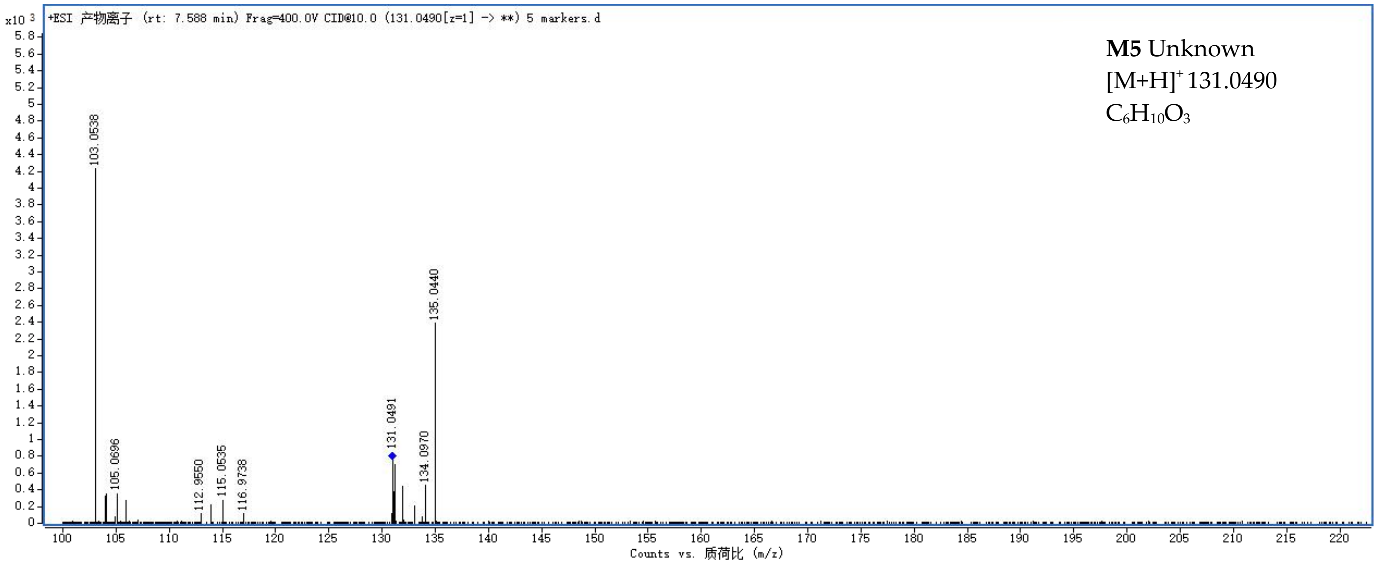
+ESI 产物离子 (rt: 7.588 min) Frag=400.0V CID@10.0 (131.0490[z=1] -> **) 5 markers.d
x10 3
M5 Unknown
[M+H]+ 131.0490
$C_6H_{10}O_3$
103.0538
105.0696
112.9550
115.0535
116.9738
131.0491
134.0970
135.0440
Counts vs. 质荷比 (m/z)

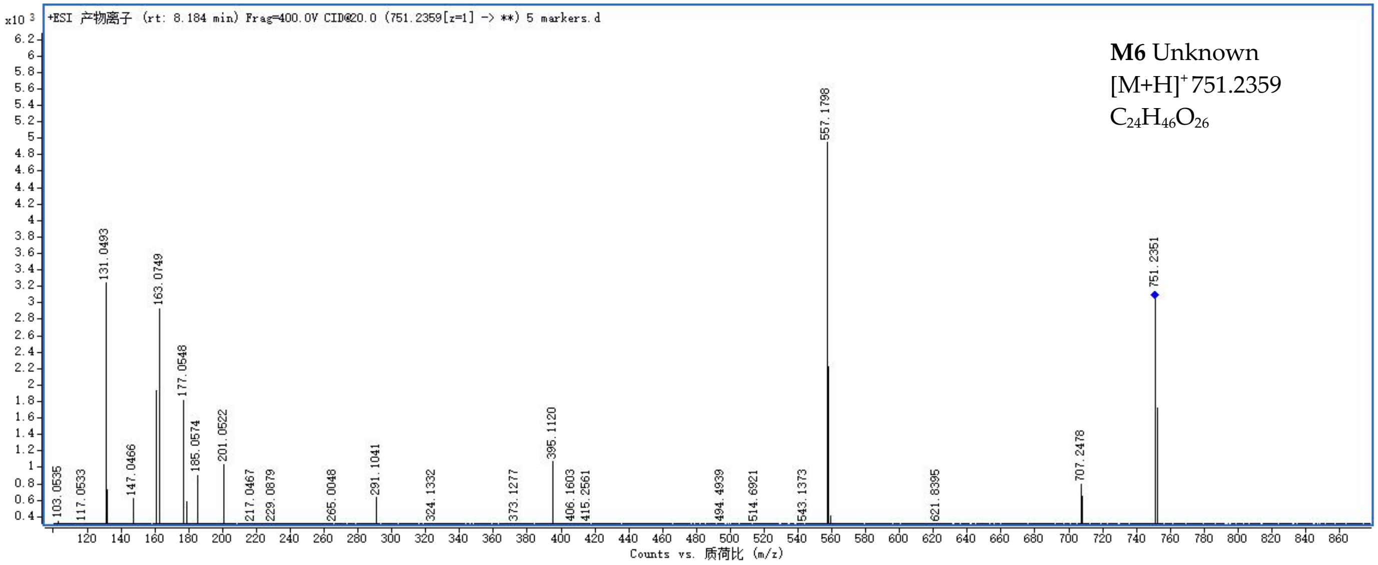
+ESI 产物离子 (rt: 8.184 min) Frag=400.0V CID@20.0 (751.2359[z=1] -> **) 5 markers.d
x10 3
M6 Unknown
[M+H]+ 751.2359
$C_{24}H_{46}O_{26}$
103.0535
117.0533
131.0493
147.0466
163.0749
177.0548
185.0574
201.0522
217.0467
229.0879
265.0048
291.1041
324.1332
373.1277
395.1120
406.1603
415.2561
494.4939
514.6921
543.1373
557.1798
621.8395
707.2478
751.2351
Counts vs. 质荷比 (m/z)

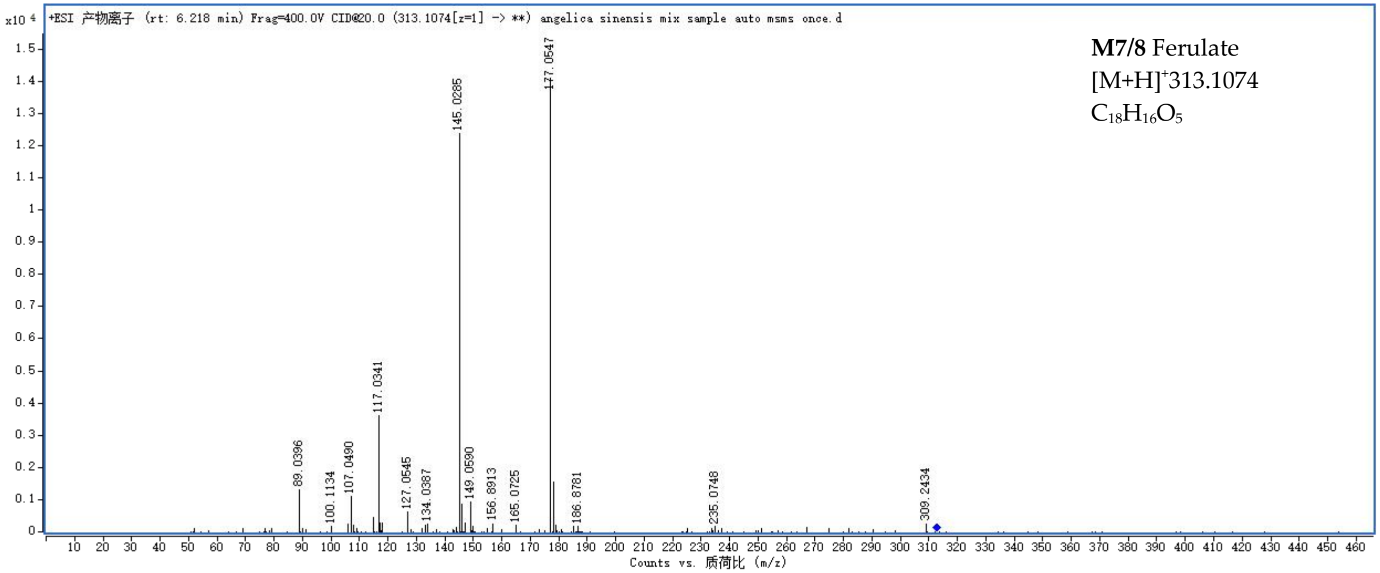
+ESI 产物离子 (rt: 6.218 min) Frag=400.0V CID@20.0 (313.1074[z=1] -> **) angelica sinensis mix sample auto msms once.d
x10 4
M7/8 Ferulate
[M+H]+313.1074
$C_{18}H_{16}O_5$
177.0547
145.0285
117.0341
89.0396
100.1134
107.0490
127.0545
134.0387
149.0590
156.8913
165.0725
186.8781
235.0748
309.2434
Counts vs. 质荷比 (m/z)

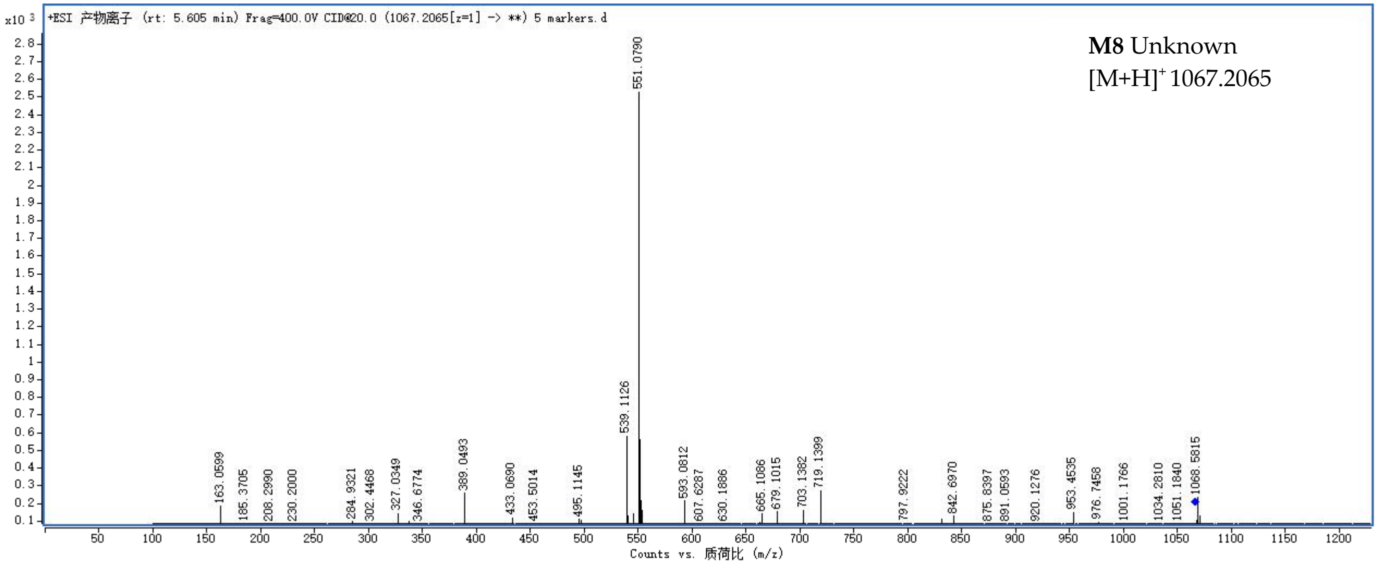
+ESI 产物离子 (rt: 5.605 min) Frag=400.0V CID@20.0 (1067.2065[z=1] -> **) 5 markers.d
x10 3
M8 Unknown
[M+H]+ 1067.2065
551.0790
539.1126
163.0599
185.3705
208.2990
230.2000
284.9321
302.4468
327.0349
346.6774
389.0493
433.0690
453.5014
495.1145
593.0812
607.6287
630.1886
665.1086
679.1015
703.1382
719.1399
797.9222
842.6970
875.8397
891.0593
920.1276
953.4535
976.7458
1001.1766
1034.2810
1051.1840
1068.5815
Counts vs. 质荷比 (m/z)

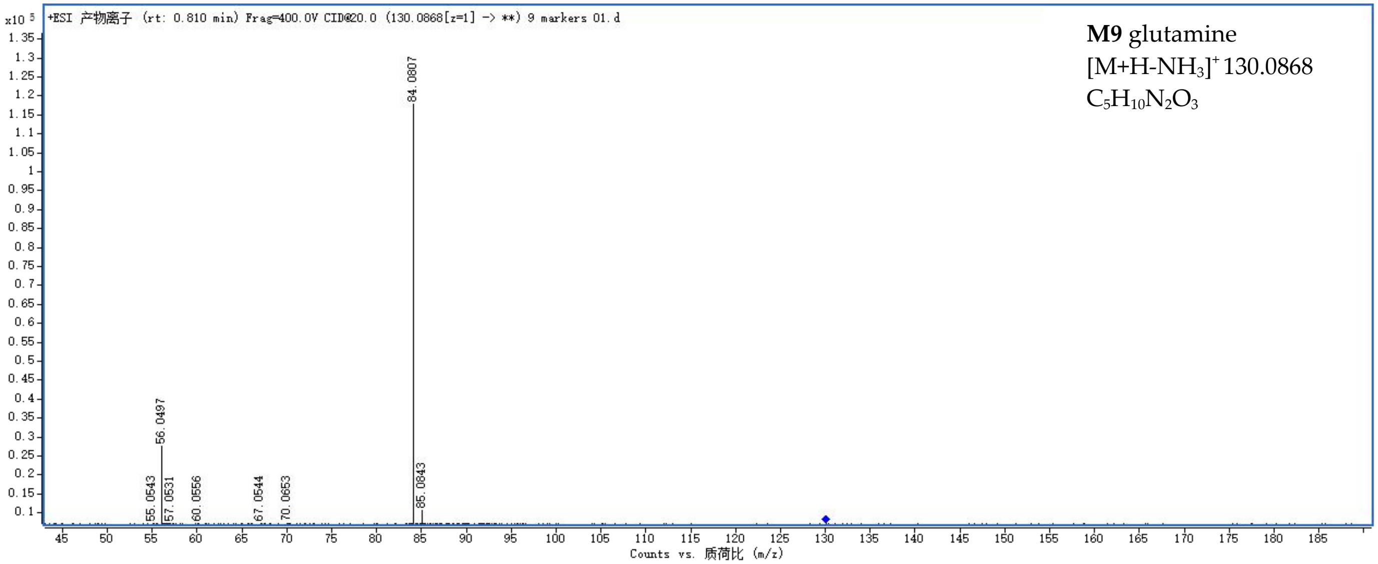

M9 glutamine
$[M+H-NH_3]^+$ 130.0868
$C_5H_{10}N_2O_3$
